# Non‐triazine photosystem II inhibitors provide effective control of metabolic atrazine‐resistant *Amaranthus tuberculatus*

**DOI:** 10.1002/ps.70766

**Published:** 2026-03-29

**Authors:** Alexander J Lopez, Ryan S Henry, Kip E Jacobs, Damilola A Raiyemo, Isabel S Werle, Patrick J Tranel

**Affiliations:** ^1^ Department of Crop Sciences University of Illinois at Urbana‐Champaign Urbana IL USA; ^2^ UPL NA Inc. Cary NC USA; ^3^ Northern Field Crops, UPL NA Inc. Cary NC USA

**Keywords:** *Amaranthus tuberculatus*, amicarbazone, atrazine, glutathione *S*‐transferase, metribuzin, molecular docking, non‐target‐site resistance, Photosystem II

## Abstract

**BACKGROUND:**

Overexpression of the Phi‐class glutathione *S*‐transferase (GST) *AtuGSTF2* has been implicated in the detoxification of atrazine in *Amaranthus tuberculatus* (Moq.) Sauer (waterhemp). However, little is known about this GST and the susceptibility of resistant populations to other Photosystem II (PSII)‐inhibitors remains unclear. Therefore, this study evaluated the efficacy of non‐triazine PSII‐inhibitors amicarbazone and metribuzin against atrazine‐resistant *A. tuberculatus*. Molecular docking analysis with these molecules was also conducted at the PSII target‐site to further investigate differences in their relative potencies and recommended field‐use rates. Furthermore, the *AtuGSTF2* gene and translated protein structure were analyzed to identify potential regulatory relationships and characterize the active site.

**RESULTS:**

Compared to atrazine, amicarbazone improved the control of metabolic atrazine resistant populations to a greater extent than metribuzin, and control was further improved when amicarbazone and metribuzin were applied in a 1.75:1 mixture. Molecular docking indicated that amicarbazone interacts with the target site similarly to atrazine and metribuzin but with greater predicted affinity. Genomic and structural analyses suggest that *AtuGSTF2* may be regulated by multiple transcription factors and the encoded protein exhibits substrate recognition promiscuity consistent with other GSTs.

**CONCLUSION:**

Together, these findings indicate that amicarbazone is a potent molecule and offers a more effective alternative to atrazine than metribuzin for managing at least some NTS atrazine‐resistant *A. tuberculatus* populations. However, tank‐mixing amicarbazone with metribuzin can provide even more effective control than either herbicide alone. Furthermore, substrate recognition promiscuity could possibly endow AtuGSTF2 with the potential for broader metabolic cross‐resistance. © 2026 The Author(s). *Pest Management Science* published by John Wiley & Sons Ltd on behalf of Society of Chemical Industry.

## Graphical abstract

Metabolism‐based atrazine‐resistant *Amaranthus tuberculatus* populations ACR and MCR exhibit little or no cross‐resistance to amicarbazone and metribuzin, suggesting that non‐halogenated Group 5 herbicides can be utilized to re‐establish effective control.

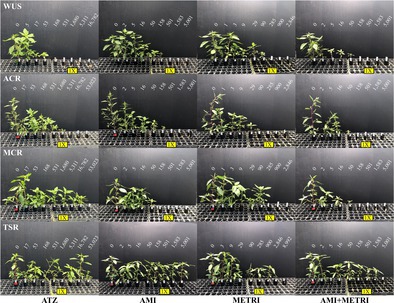

## 1 INTRODUCTION

The evolution of herbicide resistance in weedy species presents a significant challenge to modern agricultural systems reliant on chemical pest management. Among these species, *Amaranthus tuberculatus* (Moq.) Sauer (waterhemp), a dioecious summer‐annual weed, has emerged as one of the most difficult weeds to control in the midwestern United States due to widespread herbicide resistance.^1^, ^2^, ^3^ Without effective management, its rapid growth, driven by C4 carbon fixation, and prolific seed production allow it to outcompete crops, reducing corn and soybean yields by up to 74% and 56%, respectively.^4^, ^5^ To date, *A. tuberculatus* has evolved resistance to herbicides spanning seven distinct sites of action (SOAs; Groups 2, 4, 5, 9, 14, 15, and 27), with many populations now exhibiting resistance to three or more SOAs simultaneously.^6^, ^7^, ^8^, ^9^, ^10^ As a result, growers are increasingly left with fewer options for effective control of this driver weed, underscoring the urgent need to optimize existing herbicide strategies.

Group 5 PSII‐inhibitors have historically been an important class of PRE (soil‐applied) and POST (foliar‐applied) herbicides encompassing multiple diverse chemical families. Collectively, these herbicides inhibit the photosynthetic light reactions in susceptible plants by binding to the Q_B_ site in the D1 subunit of the PSII complex, thereby preventing the binding of plastoquinone and disrupting the electron flow to cytochrome b_6_f.^11^ Competition for the Q_B_ site leads to the rapid accumulation of reactive oxygen species (ROS) from excited triplet chlorophyll, ultimately causing lipid peroxidation that results in membrane damage and plant death. Among these herbicides, atrazine (triazine) has been one of the most heavily utilized PSII‐inhibitors for selective PRE and POST control of weeds in corn and sorghum systems, where natural tolerance is meditated by glutathione *S*‐transferase (GST) activity.^12^, ^13^ However, continuing atrazine use has led to resistance in several weed species, most commonly through point mutation in the plastidic *psbA* gene encoding the D1 protein, resulting in a Ser^264^Gly substitution at the target site.^14^ This mutation reduces herbicide binding to the site, thus conferring a predictable cross‐resistance pattern that renders mutant biotypes insensitive to most group 5 PSII inhibitors due to their shared binding at the Ser^264^ residue.^15^

Resistance to atrazine in *A. tuberculatus* is known to be conferred by both target‐site (TS) and non‐target‐site (NTS) resistance mechanisms.^1^ When atrazine resistance was first reported in *A. tuberculatus* in 1990, it was determined to be conferred through the common Ser^264^Gly TS resistance mutation.^16^, ^17^ Several years later, nuclear‐encoded NTS resistance was identified when atrazine resistance did not show maternal inheritance, as expected for the plastid‐encoded *psbA* gene.^18^, ^19^ In two NTS atrazine‐resistant *A. tuberculatus* populations from Adams (ACR) and Mclean (MCR) Counties in Illinois (IL), resistance was found to result from metabolic detoxification mediated by enhanced GST activity.^20^ Subsequent segregation analysis and biochemical characterization further indicated metabolic atrazine resistance in these populations was inherited as a single‐gene trait,^21^ likely caused by the overexpression of a single putative Phi‐class GST (*AtuGSTF2*).^22^ GST enzymes have been increasingly implicated to play critical roles in the detoxification of herbicides in a growing number of weeds due to their ability to catalyze the conjugation of reduced glutathione (GSH; γ‐Glu‐Cys‐Gly) to the electrophilic centers of various xenobiotic molecules, thus rendering them more soluble and enabling them to be further metabolized.^23^

Although TS resistance evolved first, the spread of resistance through this mechanism has been relatively slow, likely due to the maternal inheritance pattern of this trait combined with a notable fitness penalty caused by the mutation.^1^, ^16^ On the other hand, metabolic NTS resistance does not confer significant fitness costs in the absence of herbicide selection^24^ and can be more rapidly disseminated through pollen dispersal. Likely as a result, metabolic NTS resistance has since become the predominant mechanism of resistance to atrazine in *A. tuberculatus* and is now widespread throughout the Midwest.^25^, ^26^ Due to the often‐unpredictable nature of cross resistance that may be conferred, the rise in frequency of NTS resistance in weeds has become a major concern.^27^ However, while *A. tuberculatus* populations exhibiting NTS resistance to atrazine were reported to also be resistant to cynazine, a structurally similar triazine herbicide, multiple studies indicate that metribuzin (triazinone) still provides effective control.^19^, ^28^, ^29^ Given that atrazine metabolic detoxification results from direct GSH conjugation catalyzed by a single GST, this cross‐resistance pattern may be explained by the susceptibility of these molecules to glutathionylation.^28^ While atrazine and cynazine are both halogenated triazines with a highly electronegative chlorine necessary for nucleophilic attack by the thiol group of GSH, metribuzin and other non‐triazine PSII inhibitors do not possess a similar reactive group. Therefore, cross‐resistance beyond the triazines is not expected since direct glutathionylation is unlikely among non‐triazine PSII inhibitors without prior modification by enzymes such as cytochrome P450s.^23^

In contrast to the TS mutation conferring resistance across Group 5 herbicides, the suggested substrate specificity of this NTS mechanism opens the possibility to growers to re‐establish control using non‐triazine PSII inhibitors. However, aside from metribuzin, the susceptibility to additional non‐triazine Group 5 herbicides has not been investigated in *A. tuberculatus* populations with metabolic NTS resistance to atrazine. Therefore, the first objective of this study was to evaluate the efficacy of alternative non‐triazine PSII‐inhibitor herbicide use on NTS atrazine‐resistant *A. tuberculatus*. To accomplish this, dose–response experiments were conducted to evaluate the relative susceptibility of NTS atrazine‐resistant populations to atrazine, metribuzin, and amicarbazone. Furthermore, given the notable differences in recommended rates of active ingredients of these molecules, our second objective was to model the 3‐dimensional structure of the *A. tuberculatus* PSII D1 protein *in‐silico* and conduct molecular docking to determine whether differences in target‐site interactions could help explain the relative efficacy of the alternative non‐triazine PSII inhibitors. The GST associated with atrazine resistance has also only been identified through partial coding sequences; thus, little is known about its genetic architecture and physical structure. Further understanding the mechanistic basis of herbicide‐detoxifying enzymes will be essential to countering their influence and determining which herbicides are still effective, as well as developing new herbicides not predisposed to their influence. Therefore, our third objective was to identify the *AtuGSTF2* gene and evaluate its architecture and translated protein structure to provide a basis for its mechanism of expression and noted substrate recognition capabilities. To accomplish this, we obtained the full‐length *AtuGSTF2* from the *A. tuberculatus* reference genome, analyzed the gene and its promoter, then modeled the 3‐dimensional protein structure and analyzed its active site.

## 2 MATERIALS AND METHODS

### 2.1 Plant materials and growth conditions

In this study, two *A. tuberculatus* populations with GST‐mediated (*AtuGSTF2*) metabolic resistance to atrazine, designated ACR and MCR, were selected to evaluate the relative effectiveness of triazine and non‐triazine PSII herbicides in NTS atrazine‐resistant populations. The ACR population was developed through a greenhouse cross to select for three‐way resistance (Groups 2, 5, and 14) and was derived from a field in Adams County, IL.^8^ The MCR population was derived from an RxR cross selecting for Groups 2 and 5 resistance and originally collected from McLean County, IL.^30^ For reference, a population sensitive to atrazine, WUS, and a population resistant to atrazine through mutation of the target site, TSR, were also included. WUS is a universal sensitive population originally collected from Brown County, Ohio (OH) that is available upon request through the USDA‐ARS Germplasm Resources Information Network (GRIN) (GRIN ID: PI 698378). The TSR population was an F_2_ cross selecting for only the PSII Ser^264^Gly TS resistance mutation, derived from an atrazine‐resistant field collection from Brown County, IL crossed with a sensitive population, WCS, from Wayne County, IL.^18^

All seeds were first surface sterilized and cold‐stratified for 6 weeks at 4 °C to disrupt seed dormancy and enhance germination.^7^ Seeds were germinated in parafilm‐sealed Petri dishes lined with blotting paper soaked in 2 mL of water for 48 h in a growth chamber set for a 12/12 h photoperiod at 35/15 °C (day/night). Germinated seedlings were sown into 164 cm^3^ Cone‐tainers (Ray Leach SC10 ‘Cone‐tainer’, 31 933 Rolland Drive, Tangent, OR) filled with a sandy loam soil mix containing soil, peat, and torpedo sand (1:1:1). Slow‐release fertilizer (Osmocote® 13–13–13 slow‐release fertilizer, Scotts, 14111 Scottslawn Road, Marysville, OH) was mixed into the soil media prior to potting and subsequently added as needed. Greenhouse conditions at the University of Illinois Plant Care Facility during the experiments were set to maintain a 16/8 h photoperiod at 28/22 °C (day/night). Supplemental LED lighting (extended white, 1000 μmol m^−2^ s^−1^) was utilized during the photoperiod when average light intensity was below 600 W m^−2^ (Combo LED 600 W, C‐LED S.R.L., Via Gambellara 34, 40026 Imola (BO), Italy). Overhead shades were closed halfway when the average light intensity reached 700 W m^−2^ and were fully closed at 1000 W m^−2^.

### 2.2 Dose–response experiments

To evaluate the efficacy of using non‐triazine PSII inhibitors for controlling atrazine‐resistant *A. tuberculatus* plants with metabolic NTS resistance, dose–response experiments were conducted in the sensitive (WUS), metabolic NTS‐resistant (ACR, MCR), and TS‐resistant (TSR) populations using triazine (atrazine) and non‐triazine (amicarbazone and metribuzin) PSII inhibitors. Prior to conducting the full experiment, a sample of plants from each population (*n* = 5–7) were screened with a POST application of atrazine at 0.5X (840 g ai ha^−1^) and 5X (8400 g ai ha^−1^) field rates to confirm expected resistances. All herbicide applications were conducted using a compressed‐air research sprayer (DeVries Manufacturing, 86 956 State Highway 251, Hollandale, MN) fit with a TeeJet® 80015 EVS nozzle (TeeJet Technologies, Wheaton, IL) 46 cm above the plant canopy delivering 187 L ha^−1^ at 275 kPa. Furthermore, the same plants were also screened for the Ser^264^Gly substitution to confirm expected resistance mechanisms (see below).

After confirming expected resistance (Supporting Information, Fig. S1), additional plants from each population were grown in the same manner as described above (the experimental unit was one plant per pot). At the 4–6 cm plant height, an eight‐step, four‐fold rate titration equally spaced along a base 3.16 logarithmic scale was conducted in each population with either atrazine, amicarbazone, or metribuzin (Table 1). Furthermore, plants were also subjected to rate titration with a mixture of amicarbazone and metribuzin to evaluate whether the additive effects of these molecules can improve their individual efficacy. For this mixture, a 1.75:1 g ai L^−1^ ratio of amicarbazone to metribuzin was used, which reflects the active ingredient ratio at the recommended field rates and is representative of a commercial herbicide. While all three herbicides are often soil‐applied, herbicides were evaluated through foliar applications due to the comparable simplicity, combined with the fact that each has significant foliar activity, and the detoxifying enzyme is known to be expressed in the leaf tissue. Foliar herbicide applications were made as described above in order from lowest‐highest dose, and plants were returned to the greenhouse in a completely randomized design. Above‐ground biomass was harvested 13 days after treatment (DAT), dried for 7 days at 65 °C, and dry biomass was recorded. The experiment was repeated once in time, with three replicates per treatment (plus untreated control) in the first experimental run, and six replicates per treatment (plus untreated control) in the second experimental run. In the first run, doses of atrazine applied to the atrazine‐sensitive population (WUS) ranged from 5.3–16 800 g ai. ha^−1^ and doses applied to resistant populations (ACR, MCR, TSR) ranged from 16.8–53 000 g ai ha^−1^. In the second run, doses of atrazine applied to the ACR population were shifted down (5.3–16 800 g ai ha^−1^) to better capture the low‐dose response. Doses of amicarbazone applied alone or in combination with metribuzin to all populations ranged from 1.6–5000 g ai ha^−1^ in the first run and 0.5–1580 g ai ha^−1^ in the second run. Metribuzin doses applied alone or in combination with amicarbazone to the sensitive (WUS) and NTS‐resistant populations (ACR, MCR) ranged from 0.9–2850 g ai ha^−1^ in the first run and from 0.3–900 g ai ha^−1^ in the second run, whereas the doses applied to the TS‐resistant population (TSR) ranged from 2.9–8890 g ai ha^−1^ in the first run and from 0.9–2850 g ai ha^−1^ in the second run. All herbicide applications in both runs included crop oil concentrate (COC) at a rate of 1% v/v (Herbimax; Loveland Products, Inc., 3005 Rocky Mountain Avenue, Loveland, CO).

**Table 1:** Description of photosystem II (PSII) inhibitors and rates used in the dose–response experiments

Herbicide	Trade name	Chemical structure	Manufacturer	1X Rate	Rates used
				——g ai ha^−1^——	
Atrazine (Triazine)	Aatrex 4 L	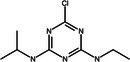	Syngenta Greensboro, NC www.syngenta.com	1680	5.3, 16.8, 53.1, 168, 531, 1680, 5310, 16 800, 53 000
Amicarbazone (Triazolinone)	Dinamic 700 WG	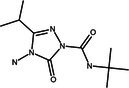	UPL, Inc. Cary, NC www.upl‐ltd.com	500	0.5, 1.6, 5, 15.8, 50.1, 158, 501, 1580, 5000
Metribuzin (Triazinone)	Metricor 75 DF	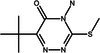	UPL, Inc. Cary, NC www.upl‐ltd.com	285	0.3, 0.9, 2.9, 9, 28.5, 90.1, 285, 900, 2850, 8890

Dose–response data analysis of the dry biomass measurements was conducted in R 4.1.1 using the *drc* 3.0.1 package to model the responses to each herbicide within each population separately.^31^, ^32^ Data were pooled across runs within each population and fit separately to a 3‐parameter log‐logistic non‐linear regression model where *y* represents the response, *x* represents the herbicide dose, *d* represents the estimated upper response limit (lower limit fixed at 0), *e* represents the estimated effective dose resulting in a 50% biomass reduction (ED_50_) between the upper and lower limits, and *b* represents the shape parameter proportional to the slope in the inflection point (ED_50_) (Eqn 1):

(1) $$y=\frac{d}{1+\exp\left(b\left(\log\left(x\right)-\log\left(e\right)\right)\right)}$$

To make comparisons among herbicides within each population from a practical field‐use standpoint, models were fit to doses normalized to the recommended field rates for each herbicide. A population was considered completely controlled when application of the herbicide at the recommended field rate resulted in 100% biomass reduction compared to untreated controls. Relative potencies (RP) of amicarbazone, metribuzin, and the mix of amicarbazone and metribuzin compared to atrazine were calculated within each population by dividing the ED_50_ estimated for atrazine (*ED*_*A*_) by the ED_50_ estimated for each herbicide (*ED*_*B*_), respectively (Eqn (2))^33^:

(2) $$RP_{B}=\frac{ED_{A}}{ED_{B}}$$

### 2.3 Ser^264^Gly TS resistance screening

Samples were screened for the Ser^264^Gly codon substitution in the *psbA* gene encoding the PSII D1 protein using a cleaved amplified polymorphic sequences assay adapted from Schultz *et al*.^25^ DNA was extracted from flash frozen leaf tissue following the CTAB protocol by Doyle and Doyle,^34^ and a fragment of the *psbA* gene around the Ser^264^ residue (~400 bp) was amplified via polymerase chain reaction (PCR) using primers and reaction conditions described by Foes *et al*.^17^ Amplified PCR products were purified using the GeneJET PCR Purification Kit (Thermo Fisher Scientific, 81 Wyman St., Waltham, MA 02454) according to manufacturer protocols, and 100 ng was digested for 8 h at 37 °C in a 20 μL reaction containing 1 μL *Bfa*I restriction enzyme (R0568S, 2.5 units μL^−1^) and 2 μL 10X Cutsmart Buffer (New England Biolabs, 240 County Road, Ipswich, MA). An aliquot of 10 μL digested product was separated on 2% agarose gel stained with SYBR safe DNA Gel Stain (Thermo Fisher Scientific) for 55 min at 120 V and visualized under ultraviolet light. Sensitive individuals contain two *Bfa*I cut‐sites and result in three bands, while only two bands are seen in resistant individuals due to a disrupted cut‐site produced from the mutation.

### 2.4 Molecular modeling

To investigate the interaction of PSII herbicides with the PSII target site and the GST conferring atrazine resistance, the 3‐D *A. tuberculatus* protein structures of PSII D1 and AtuGSTF2 proteins were modeled *in‐silico* and ligand binding was explored. The coding sequence of *AtuGSTF2* (654 bp) used for molecular modeling was obtained from the most recently available *A. tuberculatus* reference genome at the time of this study,^35^ while the sequence for the *psbA* gene (1062 bp) was obtained from a previous draft assembly due to the fragmentation of this gene in the most recent version.^36^ The *A. tuberculatus psbA* and *AtuGSTF2* sequences, respectively, were located through a local BLASTn search by querying an *Arabidopsis thaliana psbA* homolog (NCBI Reference Sequence: NC_000932.1, Gene ID: 844802)^37^ and partial coding sequences of *AtuGSTF2* (GenBank Accessions: KY196994.1, KY196995.1, KY196996.1).^22^ Conserved GST domains were analyzed using the NCBI Conserved Domain Database (CDD)^38^ and protein subcellular localization was predicted using WoLF PSORT.^39^

Protein structures were modeled using the template‐based homology‐modeling approach implemented in SWISS‐MODEL.^40^ This approach leverages the evolutionary structural conservation between homologous proteins using experimentally solved structures of high‐quality proteins with significant sequence similarity to the target protein as templates to generate accurate 3‐D structural models. The *A. tuberculatus* PSII D1 protein was modeled using a crystal structure template of PSII determined through X‐ray diffraction at a resolution of 3.2 Å from the cyanobacterium *Thermosynechococcus elongatus* (PDB: 4V82).^41^ This structure was uniquely co‐crystallized with the triazine herbicide terbutryn in the Q_B_ pocket of the D1 protein and thus was selected as an ideal template for modeling the interaction of similar herbicides given the high degree of conservation between plant and cyanobacterium D1 proteins. Apart from examples in *Zea mays* however, no Phi‐class GST proteins have been co‐crystallized with triazine herbicides in dicots. Instead, the AtuGSTF2 protein was modeled using an *A. thaliana* GSTF2 template co‐crystalized with an acetamide herbicide through X‐ray diffraction at a resolution of 2.6 Å (PDB: 1BX9).^42^ Template reliability was checked through Global Model Quality Estimates (GMQE) considering target‐template alignment and template quality. Model quality was assessed using the Distance Constraint Qualitative Model Energy Analysis (QMEANDisCo) scoring function and bond angles were checked through Ramachandran plots. Models were visualized in UCSF Chimera X 1.8^43^ and root mean square deviations (RMSD) of backbone alpha carbon (Cα) atoms were calculated based on 3‐D protein alignment to the template.

Ligand binding sites and pocket volumes were predicted using the template‐free machine learning tool P2Rank.^44^ This tool evaluates potential ligand binding sites using a trained Random Forest classifier to score the geometric and physical properties of points along the protein's solvent accessible surface (SAS) and points with high binding potential are clustered to identify likely pockets. Conservation of the predicted ligand binding sites were evaluated through multiple sequence alignment of the primary amino acid peptide sequence from the template to the target protein using Clustal Omega 1.2.4^45^ and visualized using ESPript 3.0.^46^ For further comparison, the peptide sequence of a homologous *Z. mays* Phi‐class GST (GST1) isoform, whose structure was co‐crystalized with a bound atrazine‐GSH conjugate (PDB: 1BYE),^42^ was also aligned to the target GST.

Computational molecular docking analysis of the *A. tuberculatus* D1 protein was conducted with the AutoDock suite protocol adapted from Forli *et al*.^47^ Chemical structures of the herbicide ligands atrazine (PubChem ID: 2256), amicarbazone (PubChem ID: 153920), and metribuzin (PubChem ID: 30479) were retrieved from the PubChem database (https://pubchem.ncbi.nlm.nih.gov/) and converted to PDB format with Open Babel 3.1.1.^48^ For positive control, the herbicide terbutryn (PubChem ID: 13450) co‐crystallized in the template 1BX9 was used, while glyphosate (PubChem ID: 3496) was used for negative control. To prepare structures for docking, coordinate files for the protein receptor and herbicide ligands were protonated and charges were added using AutoDockTools 1.2.4.^49^ Molecular docking analysis was performed across 20 runs for each ligand guided to the Q_B_ binding pocket of the D1 protein using AutoDock Vina 1.2.3.^50^ Predicted binding energies for the top 9 best ligand poses per run for each ligand were plotted in R and analyzed using a one‐way ANOVA, means were separated using Tukey's HSD.^31^

### 2.5 *AtuGSTF2* promoter analysis

To investigate the genetic regulation of the *AtuGSTF2* gene conferring resistance to atrazine, a regulatory prediction analysis was conducted using the promoter region directly upstream (1‐kb) of the *AtuGSTF2* transcription start site. Conserved cis‐acting regulatory elements in the promoter region were identified using the PlantCARE database.^51^ Transcription factor (TF) motifs from *A. thaliana* were also utilized to predict potential regulatory relationships using the PlantRegMap tool from the Plant Transcription Factor Database (PlantTFDB).^52^

## 3 RESULTS

### 3.1 Dose–response analysis

**Figure 1:**
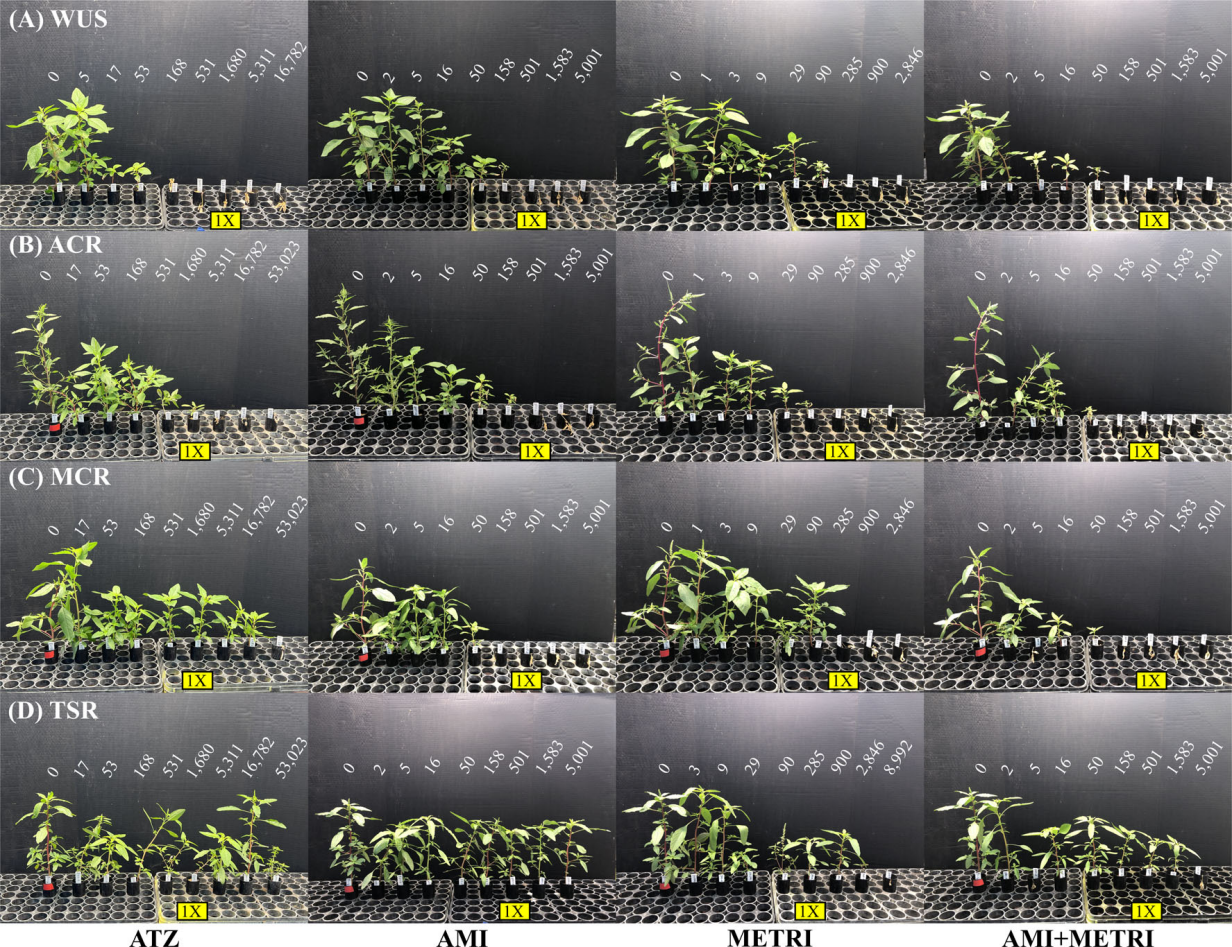
Phenotypic responses across treatment levels 13 days after treatment for each population and herbicide evaluated in the first run: WUS (A), ACR (B), MCR (C), TSR (D), atrazine (ATZ, column 1), amicarbazone (AMI, column 2), metribuzin (METRI, column 3), amicarbazone/metribuzin mix (AMI + METRI, column 4). Herbicide rates increase left–right beginning with untreated control; rates are labeled above in g ai ha^−1^ and yellow boxes indicate recommended 1X field rates.

**Figure 2:**
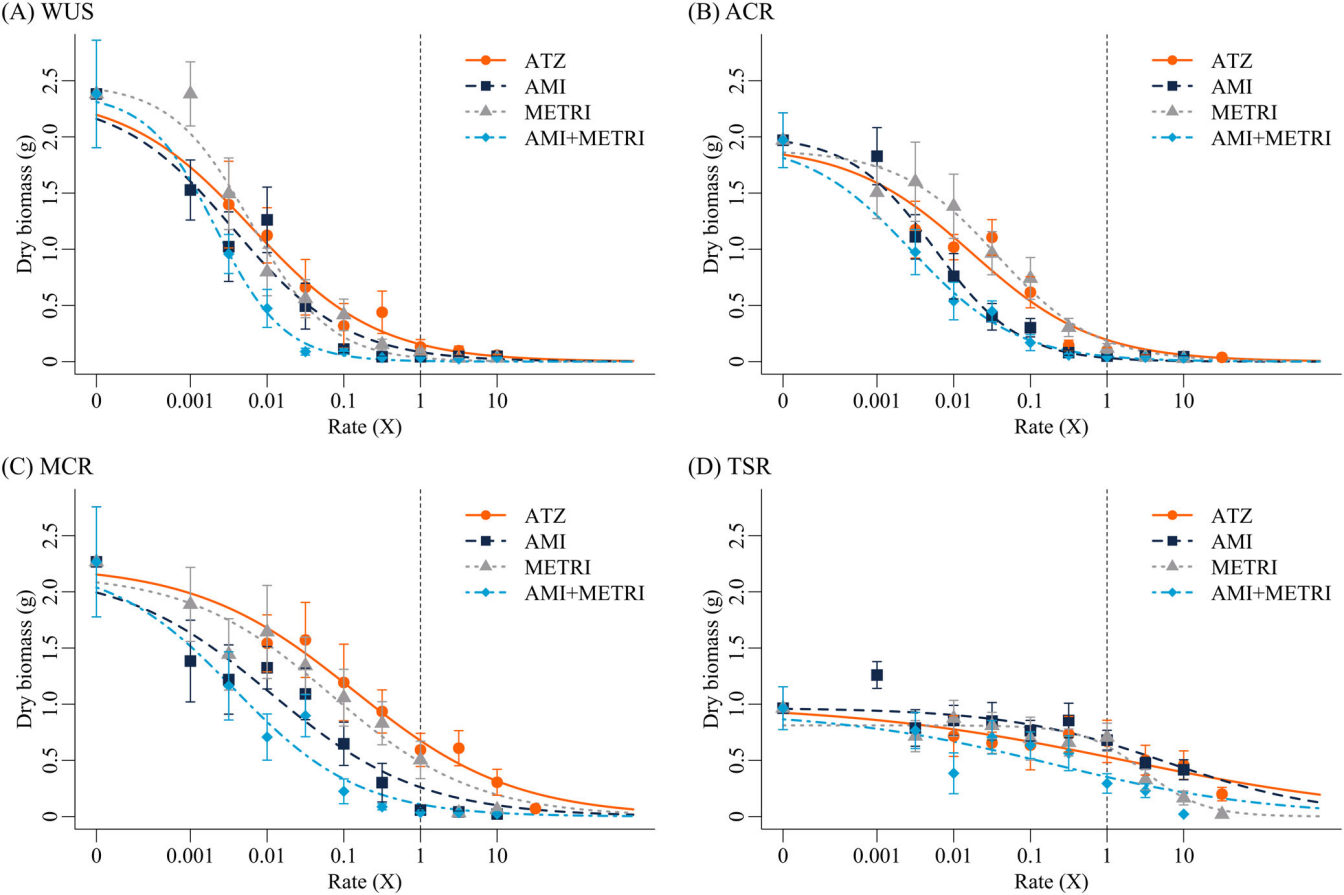
Dose–response curves modeling percent biomass reduction 13 days after treatment in response to increasing herbicide rates of atrazine (ATZ), amicarbazone (AMI), metribuzin (METRI), or a 1.75:1 mix of amicarbazone and metribuzin (AMI + METRI) across the populations WUS (A), ACR (B), MCR (C), and TSR (D). Herbicide rates are expressed relative to the recommended 1X field application rate listed in Table 1. The dashed grey line indicates the 1X field rate and each point represents the mean (± standard error) at the specified dose.

All three PSII herbicides and the amicarbazone‐metribuzin mixture achieved complete control (100% biomass reduction) of the sensitive WUS population below the recommended field rates (Table 2; Supporting Information, Tables S1 and S2; Figs 1(A) and 2(A)). However, atrazine failed to achieve complete control in the ACR and MCR populations at the recommended field rate (1680 g ai ha^−1^) (Table 2; Supporting Information, Tables S1 and S2; Figs 1(B), (C) and 2(B), (C)). In ACR, complete control was nearly achieved for atrazine at the field rate, however while severely stunted, multiple plants still survived and began to grow new healthy tissue. Less stunting was observed in response to atrazine in MCR and many plants survived at the 10X field rate. Conversely, both amicarbazone and the mixture of amicarbazone and metribuzin achieved complete control of these populations at their respective field rates. However, metribuzin applied alone achieved complete control at the recommended field rate only for ACR, while some MCR plants survived the field rate with severe stunting. In contrast, the TSR population was not completely controlled by any herbicide applied alone or in the mixture, thus limiting our ability to accurately model the response in this population (Table 2; Supporting Information, Tables S1 and S2; Figs 1(D) and 2(D)).

**Table 2:** Effective dose estimates resulting in 80% biomass reduction (ED_80_) 13 days after treatment in the populations WUS, ACR, MCR, and TSR in response to treatment with atrazine (ATZ), amicarbazone (AMI), metribuzin (METRI), or the amicarbazone/metribuzin mix (AMI + METRI)

Population	Herbicide	ED_80_ (95% CI)	ED_80_ (95% CI)	RP (95% CI)	*P*‐value
		—g ai ha^−1^—	—X Field rate—		
WUS	ATZ	146 (−46.2, 338)	8.68e‐02 (−2.75e‐02, 0.201)	—	—
	AMI	19.6 (−2.25, 41.5)	3.92e‐02 (−4.48e‐03, 8.29e‐02)	2.22 (−1.61, 6.04)	0.532
	METRI	9.17 (−0.433, 18.8)	3.22e‐02 (−1.52e‐03, 6.58e‐02)	2.70 (−1.85, 7.25)	0.462
	AMI + METRI	4.45 (0.348, 8.54)	8.88e‐03 (6.95e‐04, 1.71e‐02)	9.78 (−5.94, 25.5)	0.273
ACR	ATZ	378 (22.6, 733)	0.225 (1.35e‐02, 0.436)	—	—
	AMI	16.2 (2.76, 29.5)	3.22e‐02 (5.51e‐03, 5.90e‐02)	6.97 (−1.77, 15.7)	0.180
	METRI	81.9 (14.5, 149)	0.287 (5.07e‐02, 0.524)	0.782 (−0.195, 1.76)	0.661
	AMI + METRI	12.8 (0.680, 24.9)	2.56e‐02 (1.36e‐03, 4.98e‐02)	8.78 (−2.94, 20.5)	0.192
MCR	ATZ	6.17e+3 (−4.29e+3, 1.66e+4)	3.67(−2.55, 9.90)	—	—
	AMI	127 (−69.5, 323)	0.253 (−0.139, 0.645)	14.6 (−18.9, 48.0)	0.425
	METRI	383 (−200, 966)	1.34 (−0.703, 3.39)	2.72 (−3.49, 8.92)	0.587
	AMI + METRI	25.3 (−10.4, 61.1)	5.06e‐02 (−2.07e‐02, 0.122)	73.0 (−87.9, 234)	0.379
TSR	ATZ	8.93e+5 (−3.51e+6, 5.30e+6)	531 (−2.09e+03, 3.15e+03)	—	—
	AMI	5.64e+4 (−2.66e+5, 3.79e+5)	113 (−532, 757)	3.04 (−23.1, 29.2)	0.878
	METRI	3.05e+3 (−2.37e+3, 8.47e+3)	10.7 (−8.31, 29.7)	48.8 (−205, 302)	0.711
	AMI + METRI	8.47e+3 (−2.21e+4, 3.90e+4)	16.9 (−44.1, 77.9)	29.6 (−150, 209)	0.755

X field rate denotes the herbicide dose expressed as a multiple of the recommended field rate (1X). The rate listed for the mixture (1.75:1 amicarbazone:metribuzin) reflects the rate for amicarbazone only. Relative potencies (RP) are calculated by dividing the ED_80_ of atrazine by the ED_80_ of the herbicide in each population, using ED_80_ values relative to the 1X field rate.

Compared to atrazine, there was a consistent trend of amicarbazone exhibiting greater relative potency in both NTS atrazine‐resistant populations when normalized to the 1X field rate. At the estimated ED_80_ dose, relative potency values were 6.97 and 14.6 for ACR and MCR, respectively (Table 2). A more similar response compared to atrazine was observed for metribuzin, which exhibited a slightly lower relative potency when normalized to the 1X field rate in ACR but slightly greater relative potency in MCR. For ACR, relative potency at the estimated ED_80_ dose was 0.782, whereas relative potency was 2.72 in MCR (Table 2). When applied as a mixture, amicarbazone plus metribuzin exhibited greater relative potency compared with atrazine that was higher than either herbicide alone. Relative potency at the estimated ED_80_ dose was 8.78 and 73.0 for ACR and MCR, respectively. Despite the trend of greater potency of both amicarbazone and the mixture, relative potency estimates for all treatments at the estimated ED_80_ doses were not significantly different from 1 (α = 0.05) due to notable variability in the observed responses.

### 3.2 PSII D1 molecular docking

**Figure 3:**
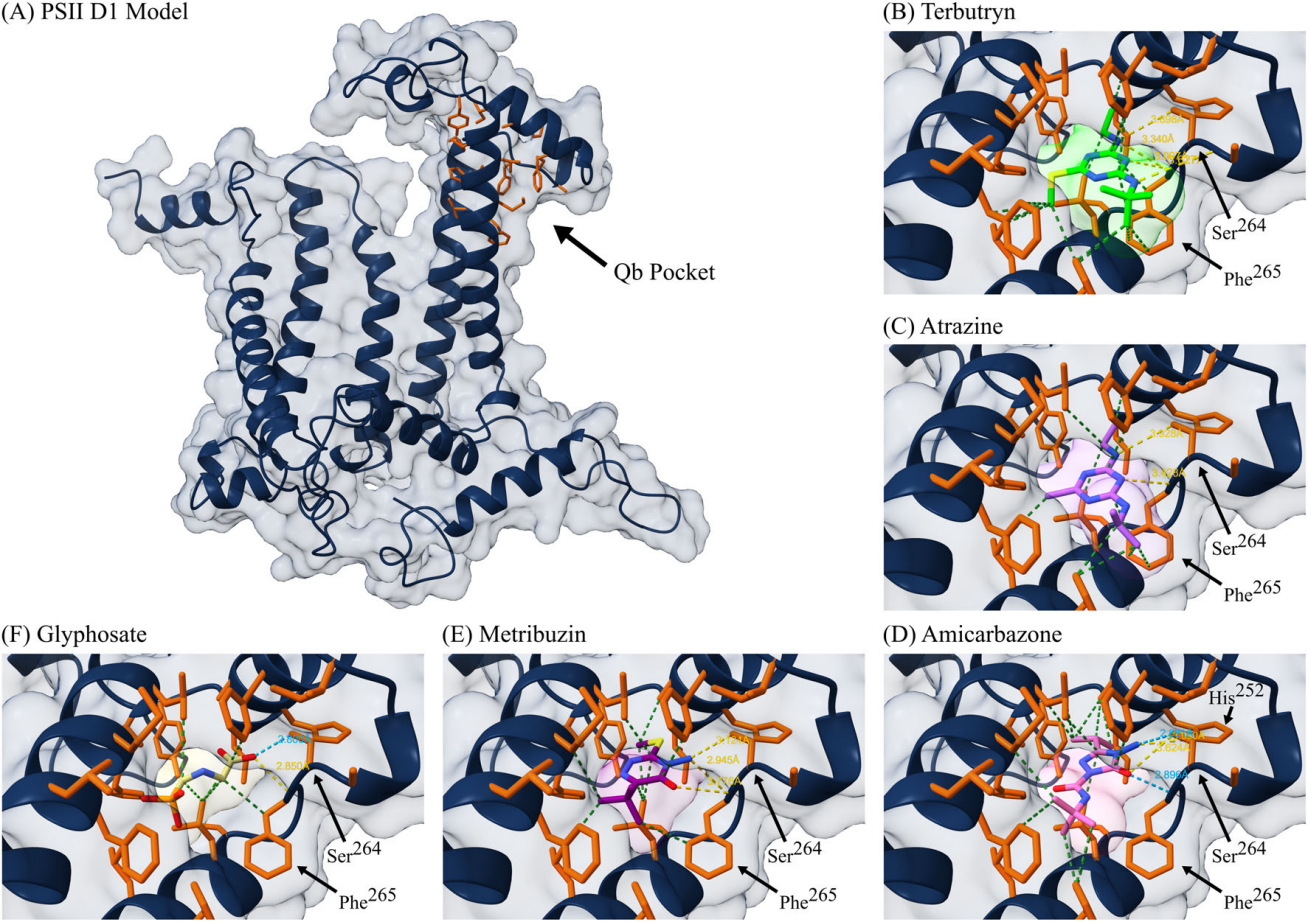
Homology‐modeled *Amaranthus tuberculatus* PSII D1 protein (A) and the top docking poses for terbutryn (B), atrazine (C), amicarbazone (D), metribuzin (E), and glyphosate (F). Residues predicted to form the binding pocket are shown in orange, hydrogen bonds are depicted as blue (strict) or yellow (relaxed) dashed lines, and Van der Waals contacts are depicted as green dashed lines.

Overall, the peptide sequence and molecular structure were well conserved with minimal stereochemical differences between the modeled *A. tuberculatus* PSII D1 protein and *T. elongatus* D1 crystal structure template (Supporting Information, Table S3, Supporting Information, Fig. S2). The global QMEANDisCo composite score of the model was also moderately high, indicating sufficient quality for the predicted structure to be suitable for *in‐silico* molecular docking. All three PSII herbicides consistently docked within the Q_B_ pocket with their reactive groups oriented towards the Ser^264^ residue (Fig. 3). Amicarbazone (−7.4 kcal mol^−1^) had the lowest binding energy, and thus greatest binding affinity, among the top‐ranked binding poses predicted for each herbicide, followed by metribuzin (−6.5 kcal mol^−1^), and then atrazine (−6.3 kcal mol^−1^) (Table 3, Supporting Information, Fig. S3). At least two conserved hydrogen bonds, one to the hydroxyl group at Ser^264^ and one to the amide backbone at Phe^265^, were possible among all three molecules. At Ser^264^, hydrogen bonds are likely to be formed between the ethyl‐amino group of atrazine and amino groups of amicarbazone and metribuzin. At Phe^265^, hydrogen bonds are likely to be formed between N5 of the atrazine ring and the keto groups of amicarbazone and metribuzin. In the case of amicarbazone, additional hydrogen bonds between the keto group and Ser^264^, as well as between the amino group and His^252^ seem possible. Furthermore, a greater number of hydrophobic contacts and Van der Waals interactions seem to contribute to its stabilization. For metribuzin, an additional hydrogen bond between the amino group and the acyl group of Phe^265^ could also be possible. Terbutryn docked in a similar orientation to that observed in the crystalized template, with minor deviations among atoms involved in hydrogen bonding (RMSD = 0.776), supporting the reliability of the docking results. Furthermore, glyphosate (−4.3 kcal mol^−1^) exhibited binding energy +2 kcal mol^−1^ higher than atrazine, indicating little to no competitive binding affinity for this site relative to the PSII inhibitors.

**Table 3:** Summary of predicted binding energies for herbicides docked within the plastoquinone‐B (Q_B_) pocket of the *Amaranthus tuberculatus* PSII D1 homology model

Ligand	Top pose	Mean*	Standard deviation
	—— Binding energy (kcal/mol) ——		
Amicarbazone	−7.358	−5.942^a^	0.697
Metribuzin	−6.455	−5.426^b^	0.425
Atrazine	−6.298	−4.926^c^	0.592
Terbutryn	−6.018	−5.020^c^	0.495
Glyphosate	−4.258	−3.543^d^	0.299

Binding energies are reported for the top‐ranking pose and as the average across 20 molecular docking simulations for each herbicide.

* Means with shared letters are not significantly different (α = 0.05).

### 3.3 AtuGSTF2 in‐silico analysis

**Figure 4:**
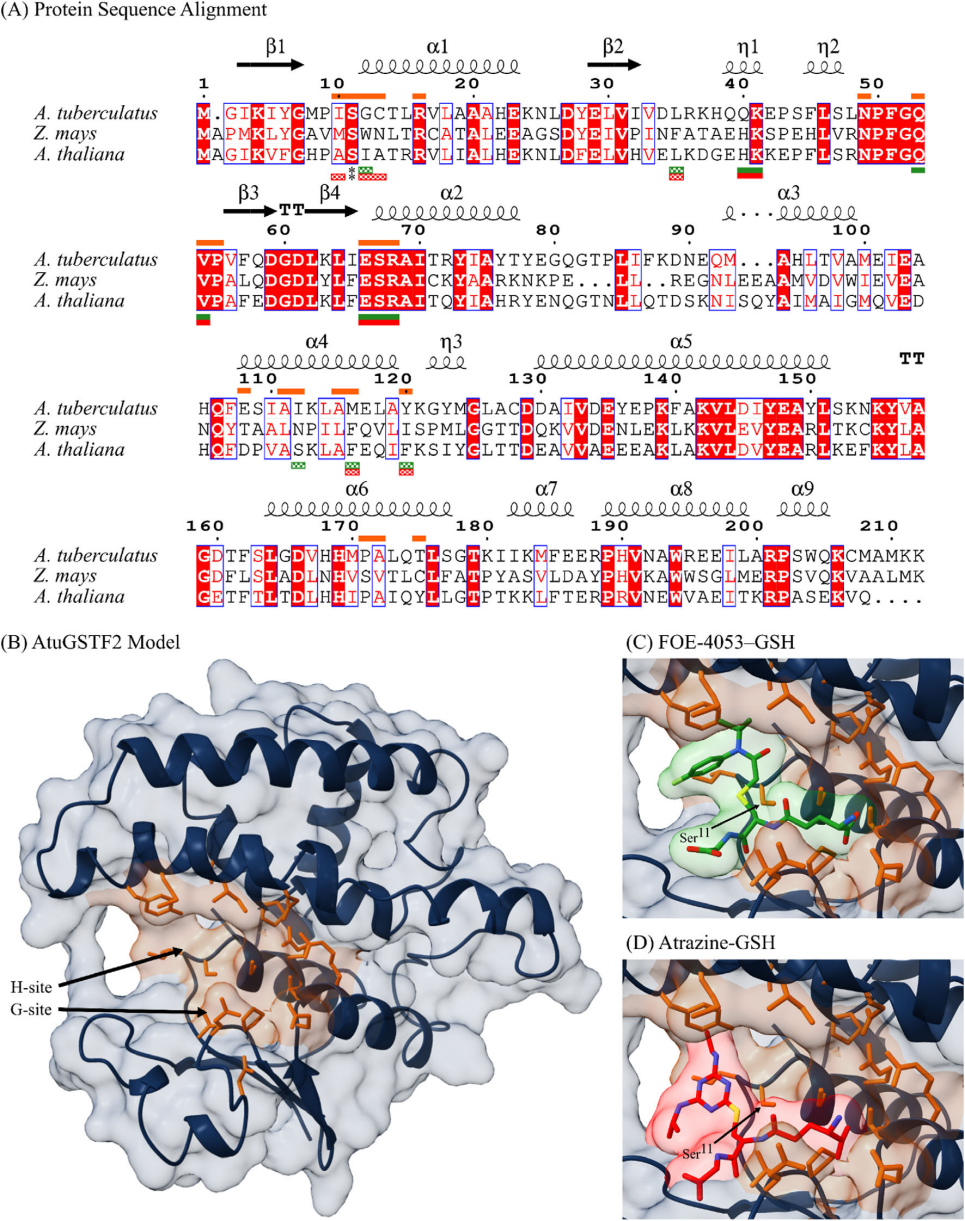
Multiple sequence alignment of *Amaranthus tuberculatus* (AtuGSTF2), *Zea mays* (1BYE), and *Arabidopsis thaliana* (1BX9) Phi‐class GSTF2 proteins (A). The secondary protein structure of AtuGSTF2 is depicted above the sequence and residues predicted to form the active site binding pocket are indicated by orange bars above the sequence. Residues experimentally determined to form the active sites of *A. thaliana* and *Z. mays* GSTs are highlighted in green and red bars, respectively, below the sequence; G‐site residues are indicated by solid bars, H‐site residues are indicated by checkered bars, and the catalytic residue is indicated by an asterisk. Residues in white font with red highlight are strictly conserved and those in red font share similar biochemical properties. Homology modeled AtuGSTF2 protein (B) and superimposed crystalized herbicide‐glutathione conjugates of FOE‐4053‐GSH (1BX9) from *A. thaliana* (C) and atrazine‐GSH (1BYE) from *Z. mays* (D); residues predicted to form the binding pocket are shown in orange.

The gene best matching the queried *AtuGSTF2* partial coding sequences (96.7% sequence identity) was identified on chromosome 16 (Chr16:4416947–4419849) of the reference genome. This gene (AmaTu_RefChr16g245410, 2902 bp) is annotated with three exons (654 bp) and is translated into a 218 amino acid peptide (24.656 kDa) predicted to be localized to the plasma membrane and cytosol. Analysis of conserved GST N‐ and C‐terminal domains combined with BLASTp query using the protein sequence confirmed the classification as a Phi‐class GST. Subsequent promoter analysis revealed several predicted cis‐regulatory elements and indicated potential regulatory relationships among 21 TFs from seven families (TCP, Dof, MYB, E2F/DP, ARF, B3, and NAC) (Supporting Information, Table S4 and Fig. S4). In addition to *AtuGSTF2*, three other Phi‐class GST genes were identified within a 30 kbp surrounding region. The sequences of these GST genes ranged in similarity from 51.9–60% compared to *AtuGSTF2* and from 55.5–82.1% amongst one another, suggesting possible tandem duplication events (Supporting Information, Fig. S5). Furthermore, another Phi‐class GST gene with 74.1% similarity to *AtuGSTF2* was identified approximately 7 Mbp downstream on chromosome 16, suggesting a possible segmental duplication event. Despite only moderate sequence similarity, the overall molecular structure of the predicted AtuGSTF2 protein was well conserved compared to the *A. thaliana* template (Supporting Information, Table S3). The protein active site was predicted to be formed by two overlapping binding pockets in the cleft between the N‐ and C‐terminal domains, together consisting of 21 residues surrounding the catalytic Ser^11^ residue (Supporting Information, Table S5). These residues aligned directly with 4/6 and 5/6 residues forming the G‐ and H‐sites in *A. thaliana*, and to 5/7 and 4/5 residues forming the G‐ and H‐sites in *Z. mays*, respectively (Fig. 4). Residues aligned to the G‐sites of these species were conserved, while all residues aligned to the H‐sites differed. Superimposition of the conjugated inhibitor from the *A. thaliana* (acetamide) and *Z. mays* (atrazine) templates in the active site of AtuGSTF2 did not reveal any significant steric hinderance, indicating possible promiscuity in substrate binding.

## 4 DISCUSSION

### 4.1 PSII inhibitor efficacy

In this study, we evaluated the efficacy of non‐triazine PSII inhibitors for controlling *A. tuberculatus* with metabolic atrazine (triazine) resistance. As anticipated, the sensitive WUS population could be completely controlled by all three herbicides below the recommended field rates. Conversely, the TSR population was not completely controlled by any herbicide, which limited our ability to accurately estimate ED values in this population. Atrazine did not completely control the NTS resistant populations at the recommended field rate; however, the estimated ED_50_ values for ACR and MCR were lower than initially indicated in these populations.^8^, ^21^ This may be explained by the fact that these previous studies applied their treatments to plants that were two to three times larger, which would lead to more robust resistance observations. Nonetheless, several plants still survived at the recommended field rate (1680 g ai ha^−1^) in both populations. Despite reports indicating similar rates of atrazine metabolism and expression levels of the detoxifying enzyme (AtuGSTF2) in ACR and MCR,^20^, ^22^ MCR plants also survived up to 10X the field rate while ACR plants did not. A differential response between these populations was previously indicated when atrazine was applied PRE or in combination with the GST inhibitor NBD‐Cl.^53^ Compared to ACR, Ma *et al*.^53^ previously showed that the MCR population was more resistant to atrazine when applied alone and the level of resistance was also less impacted when applied with the inhibitor. In MCR, NTS resistance to Group 27 herbicides has been reported to be associated with metabolic atrazine resistance, thus it is possible that additional NTS resistance genes may contribute to enhance atrazine resistance relative to ACR.^54^, ^55^

Previous reports indicated that the estimated ED_50_ values in response to POST‐applied metribuzin in populations segregating for NTS atrazine resistance (44 g ai ha^−1^) were similar to uniformly sensitive populations (32 g ai ha^−1^).^19^ These values are comparable to the ED_50_ values reported in this study and were consistently lower than atrazine on a g ai ha^−1^ basis. However, relative to atrazine when normalized to the field rate, the potency of metribuzin was not vastly greater in either NTS resistant population, and complete control in MCR was only achieved at rates greater than the recommended field rate. Metribuzin is more commonly applied PRE for controlling broadleaf weeds in soybean fields.^56^ When applied PRE, metribuzin (560 g ai ha^−1^) was reported to completely control *A. tuberculatus* populations with metabolic atrazine resistance from East Nebraska.^26^ Similarly, Westerveld *et al*.^29^ reported that metabolic atrazine‐resistant populations from Canada were effectively controlled by 88% and 95% when metribuzin was applied PRE at 560 and 1200 g ai ha^−1^, respectively. Conversely, metribuzin applied POST in the same study provided only 65% and 70% control at the same rates and required a significantly higher dose to achieve 50% control (245 g ai ha^−1^) than when applied PRE (133 g ai ha^−1^). Metribuzin is thus considered to be less efficacious for controlling metabolic atrazine resistant *A. tuberculatus* when applied POST *vs* PRE. However, it should be noted that these studies applied rates two to four times higher than what was considered the 1X rate in this study (285 g ai ha^−1^), which provided much greater biomass reduction here and would have been considered controlled regardless of application timing in those studies. Nonetheless, metribuzin did completely control the sensitive WUS population despite being applied POST and reduced biomass by 50% at a much lower rate compared to the sensitive populations used in the study by Patzoldt *et al*.^19^ One explanation for this could be the fact that this population is of the *tuberculatus* variety rather than the *rudis* variety, and is a non‐agricultural population that is likely less adapted to deal with the general effects of abiotic stress.^57^, ^58^ Interestingly, while metribuzin did not completely control the TSR population, it did result in a notable reduction in biomass relative to atrazine and amicarbazone at rates above the field rate, which was further observed in the mixture with amicarbazone. While this is contrary to much of the literature, this may indicate that metribuzin could possibly bind the target‐site in different ways when the Ser^264^ residue is substituted, as suggested in *Pisum sativum*,^59^ but further experiments are needed to confirm this.

Among the PSII‐inhibitors evaluated, when applied alone, amicarbazone provided complete control of the NTS resistant populations ACR and MCR at the lowest rates, both in terms of g ai ha^−1^ and when normalized to the 1X field rate. Although not statistically significant due to variability in the responses, amicarbazone consistently exhibited a greater relative potency compared to atrazine than was observed for metribuzin. Molecular docking to the PSII D1 target site indicated that the amicarbazone molecule interacted with the Q_B_ pocket in a similar manner to atrazine and metribuzin as previously suggested,^60^ but a greater predicted binding affinity was observed, likely resulting from additional hydrophobic contacts further stabilizing the molecule. Consistent with our findings, amicarbazone was previously reported to inhibit photosynthetic electron transport more rapidly and inhibit oxygen evolution to a greater extent than atrazine, indicating a greater potency of the molecule.^60^ However, the indication of increased affinity for the target‐site was not evident compared to metribuzin in the sensitive WUS population. This could be due to heightened sensitivity of the variety but also may suggest that there are additional factors that influence the observed response that could be improved in future amicarbazone formulations, such as foliar uptake. Although amicarbazone is a relatively recently introduced PSII‐inhibitor that has been primarily utilized in sugarcane^61^ and turfgrass^62^, ^63^ systems, it is also being developed as a potent alternative for preemergence weed control in corn.^64^ Like other PSII‐inhibitors, amicarbazone did not completely control individuals with the Ser^264^Gly target site mutation, as was previously reported in *Poa annua* L. (annual bluegrass).^65^ However, considering the increased potency of this broad‐spectrum herbicide combined with the ability to completely control metabolic atrazine‐resistant *A. tuberculatus*, amicarbazone presents a suitable option for managing this driver weed. Amicarbazone is also reported to be highly water‐soluble, mobile in the soil, and does not dissociate when dissolved.^66^, ^67^ Furthermore, when applied PRE, the half‐life of amicarbazone was less than atrazine, but nearly twice that of atrazine in a field with enhanced microbial atrazine degradation.^64^ Therefore, PRE‐application of this herbicide would be expected to provide residual control in fields with enhanced soil degradation from the overuse of atrazine.^68^

When both amicarbazone and metribuzin were applied together as a tank mix, complete control was achieved at lower rates and the relative potency compared to atrazine was greater in both NTS‐resistant populations than when amicarbazone or metribuzin were applied alone. This improved performance relative to either herbicide alone is likely due to the additive effect of these two herbicides, which enables more active ingredients to be applied and overwhelm the target‐site while staying within the allowed application rate within the growing season of either herbicide. Given that amicarbazone controlled both NTS‐resistant populations at lower rates than metribuzin and at similar rates to the mixture in ACR, it is likely that amicarbazone contributed more to the observed response than metribuzin. Both amicarbazone and metribuzin required higher rates to control MCR than ACR, which again may be explained by additional NTS resistance towards Group 27 herbicides in MCR.^54^ Improved control of POST‐applied PSII‐inhibitors in weedy species has also been achieved through synergistic activity resulting from tank mixing with Group 27 herbicides.^69^, ^70^ Synergism between atrazine and mesotrione was reported to overcome metabolic atrazine resistance in *Abutilon theophrasti*.^71^ Furthermore, atrazine tank mixed with Group 27 herbicides injured ACR plants (Group 27 sensitive) to a greater extent than either herbicide alone, but a synergistic response in MCR (Group 27 resistant) could only be observed in combination with metribuzin.^28^, ^54^ However, the recent occurrence of Group 27 resistance in *A. tuberculatus* is likely to be intensified through continued selection, thus threatening this approach. Therefore, improved control of such populations may be more sustainable through tank mixing amicarbazone with metribuzin.

### 4.2 AtuGSTF2 in‐silico analysis

Glutathione *S*‐transferase mediated metabolic NTS resistance has been reported to herbicides across multiple SOAs in *A. tuberculatus*,^22^, ^72^, ^73^ however, few studies have identified and investigated the causal enzyme(s) responsible due to the often‐complex nature of NTS resistance. Here, the active site and promoter region of the *AtuGSTF2* gene, recently indicated to confer atrazine metabolic resistance in *A. tuberculatus*,^22^ were investigated *in‐silico* to better understand its binding capabilities and expression. Phi‐class GSTs are well known to exhibit significant diversity in the substrate‐binding H‐site that has led to variation in substrate recognition among homologs.^74^ While the H‐site of AtuGSTF2 was structurally comparable to the homologous GSTF2 protein from *A. thaliana*, residues predicted to participate in binding were not conserved, thus indicating differences in substrate specificity. Furthermore, unlike most enzymes, which recognize a narrow range of substrates, GSTs contain a relatively large and conformationally flexible active site that enables a wide range of hydrophobic molecules to bind, contributing to their characteristic functional promiscuity.^75^ The noted potential promiscuity of AtuGSTF2 is indicated by a lack of potential steric hindrance by the conjugated substrate derived from the *Z. mays* (atrazine) or *A. thaliana* (acetamide) templates when superimposed. This may suggest that this GST could be capable of accommodating a wide variety of herbicide molecules beyond the triazine herbicides, including possibly the non‐triazine PSII inhibitors evaluated in this study. Therefore, plants that develop the ability to modify these molecules into a form that can participate in the conjugation reaction could utilize this highly expressed GST to overcome their toxic effects. Given that the NTS‐resistant populations ACR and MCR were not as susceptible to metribuzin as initially hypothesized, it may be possible that these populations could be expressing enzymes such as cytochrome P450s capable of modifying metribuzin into a form that can be metabolized by this GST. Cytochrome P450s were shown to be involved in resistance to Group 27 herbicides in MCR,^20^ however, their potential involvement remains speculatory and needs to be further evaluated.

Although mutation in the active site conferring a greater specific activity towards atrazine in resistant plants is possible, plants from ACR and MCR had 1000‐fold higher expression of *AtuGSTF2* and sensitive plants still had some activity towards atrazine, albeit lower than ACR or MCR, suggesting that higher activity is due to enhanced expression.^22^ The genetic mechanism regulating the overexpression of this gene in resistant biotypes is not yet known, but higher expression in resistant plants could be caused by various factors such as mutation in cis‐ or trans‐regulatory elements, chromatin remodeling, mRNA stability, or a change in a transcription factor.^76^ Analysis of the promoter region from the atrazine‐sensitive refence genome revealed multiple hormone‐ and light‐responsive cis‐elements consistent with the diurnal regulation and hormone‐inducibility reported in other GSTs.^77^, ^78^ Potential regulatory relationships were also identified with several TFs belonging to the TCP and Dof families known to be involved in hormone signaling and abiotic stress response.^79^, ^80^ Given that *AtuGSTF2* was found to be clustered with other highly similar GSTs, it is also possible that copy number variation from further duplication could contribute to elevated expression. However, additional work comparing the genomes of resistant and susceptible plants is needed to elucidate the mechanism.

## 5 CONCLUSION

The results of this study highlight the critical importance of understanding the mechanisms of herbicide resistance within a weed population to make informed decisions on effective herbicide management strategies. Our results support the hypothesis that the detoxifying GST responsible for atrazine metabolic resistance in *A. tuberculatus* exhibits selectivity for halogenated triazine PSII‐inhibitors, maintaining a greater level of susceptibility to non‐triazine PSII‐inhibitors. In the populations investigated, amicarbazone provided complete control at lower rates compared to metribuzin when applied POST and thus presents a more efficient alternative when applied alone. However, improved control relative to either herbicide alone can be achieved through tank mixing these herbicides. Additional work is needed to further evaluate the observed additive effect of the mixture and determine whether this pattern is consistent in other populations indicated with metabolic atrazine resistance, as well as if similar control is observed when applied PRE. Furthermore, determining the genetic basis of enhanced expression of the GST conferring resistance to atrazine will require comparisons of resistant and susceptible individuals in a segregating population through genetic mapping and functional genomics using techniques such as Assay for Transposase‐Accessible Chromatin sequencing (ATAC‐seq), DNA Affinity Purification sequencing (DAP‐seq), and RNA sequencing.

## CONFLICT OF INTEREST

R.S.H. is and K.E.J. was employed at UPL NA Inc.

## AUTHOR CONTRIBUTIONS

A.J.L. conducted the experiments, performed data analysis, and drafted the original manuscript. A.J.L., R.S.H., K.E.J., and P.J.T. conceptualized the experiments. R.S.H. and P.J.T. acquired funding and supervised the experiments. A.J.L., R.S.H., K.E.J., D.A.R., I.S.W, and P.J.T reviewed and edited the manuscript.

## Supporting information


**Table S1.** Effective dose estimates resulting in 20% biomass reduction (ED_20_) 13 days after treatment (DAT) in the populations WUS, ACR, MCR, and TSR in response to treatment with atrazine (ATZ), amicarbazone (AMI), metribuzin (METRI), or the amicarbazone/metribuzin mix (AMI + METRI). X field rate denotes the herbicide dose expressed as a multiple of the recommended field rate (1X). The rate listed for the mixture (1.75:1 amicarbazone:metribuzin) reflects the rate for amicarbazone. Relative potencies (RP) are calculated by dividing the ED_20_ of atrazine by the ED_20_ of the herbicide in each population, using ED_20_ values relative to the field rate.
**Table S2.** Effective dose estimates resulting in 50% biomass reduction (ED_50_) 13 days after treatment in the populations WUS, ACR, MCR, and TSR in response to treatment with atrazine (ATZ), amicarbazone (AMI), metribuzin (METRI), or the amicarbazone/metribuzin mix (AMI + METRI). X field rate denotes the herbicide dose expressed as a multiple of the recommended field rate (1X). The rate listed for the mixture (1.75:1 amicarbazone:metribuzin) reflects the rate for amicarbazone only. Relative potencies (RP) are calculated by dividing the ED_50_ of atrazine by the ED_50_ of the herbicide in each population, using ED_50_ values relative to the field rate.
**Table S3.** Structural assessment metrics for the 3‐D molecular models of *Amaranthus tuberculatus* PSII D1 and GSTF2 proteins.
**Table S4.** Cis‐acting regulatory elements (CARE) identified in the promoter region of the *Amaranthus tuberculatus AtuGSTF2* gene.
**Table S5.** Summary of predicted substrate binding pockets at the active sites of the *Amaranthus tuberculatus* PSII D1 and GSTF2 protein homology models. Residues overlapping between predicted pockets for the AtuGSTF2 model are indicated in bold.
**Fig. S1.** Atrazine resistance prescreening across WUS, ACR, MCR, and TSR populations. (A) In‐silico PCR and amplicon restriction enzyme digestion showing the expected target‐site resistance assay banding patterns for the Ser^264^Gly mutation. (B) Agarose gel‐electrophoresis showing the target‐site resistance assay banding patterns observed in the populations evaluated; on the left is a 100‐bp ladder and on the right is a 50‐bp ladder. (C) Total damage relative to the control in response to atrazine applied POST at 0.5X (left) and 5X field rates (right).
**Fig. S2.** *Amaranthus tuberculatus* PSII D1 protein multiple sequence alignment against homologous proteins from *Arabidopsis thaliana* (7OUI), *Pisum sativum* (5XNL), *Thermosynechococcus elongatus* (4V82), and *Thermostichus vulcanus* (7CJI). Stretches of conserved residues are enclosed in a blue box, with strictly conserved (identical) residues shown in white with a red background and functionally conserved (similar properties) residues shown in red with no background; non‐conserved residues are shown in black with no background. Secondary structures of the predicted model and template model are shown above and below the alignment, respectively, and labeled with the type of structure: α (alpha helix), η (short alpha helix), β (beta sheet), and T (turn).
**Fig. S3.** Distribution of predicted binding energies (kcal/mol) reported across all 20 docking simulations for each herbicide docked within the Q_B_ pocket of the *Amaranthus tuberculatus* PSII D1 protein homology model: amicarbazone (AMI), metribuzin (METRI), atrazine (ATZ), terbutryn (TBT), glyphosate (GLYPH).
**Fig. S4.** The number of transcription factors (TF) belonging to different TF families predicted to have potential regulatory relationships with *AtuGSTF2*.
**Fig. S5.** *Amaranthus tuberculatus* AtuGSTF2 (AmaTuRefChr16g245410) protein multiple sequence alignment against the three GSTs (AmaTuRefChr16g245400, AmaTuRefChr16g245420, and AmaTuRefChr16g245440) identified within 30‐kb of AtuGSTF2 on chromosome 16. Stretches of conserved residues are enclosed in a blue box, with strictly conserved (identical) residues shown in white with a red background and functionally conserved (similar properties) residues shown in red with no background; non‐conserved residues are shown in black with no background. Secondary structure of the predicted model and template model are shown above and below the alignment, respectively, and labeled with the type of structure: α (alpha helix), η (short alpha helix), β (beta sheet), and T (turn).

## Data Availability

Data available upon reasonable request.
