# Supplementary material for: Non‐triazine photosystem II inhibitors provide effective control of metabolic atrazine‐resistant Amaranthus tuberculatus
Source: Pest Manag Sci. 2026 Mar 29;82(7):6864–76. doi: 10.1002/ps.70766 (PMC13240695; doi:10.1002/ps.70766)
Supplement: Supplementary file 1 — Table S1. Effective dose estimates resulting in 20% biomass reduction (ED20) 13 days after treatment (DAT) in the populations WUS, ACR, MCR, and TSR in response to treatment with atrazine (ATZ), amicarbazone (AMI), metribuzin (METRI), or the amicarbazone/metribuzin mix (AMI + METRI). X field rate denotes the herbicide dose expressed as a multiple of the recommended field rate (1X). The rate listed for the mixture (1.75:1 amicarbazone:metribuzin) reflects the rate for amicarbazone. Relative potencies (RP) are calculated by dividing the ED20 of atrazine by the ED20 of the herbicide in each population, using ED20 values relative to the field rate. Table S2. Effective dose estimates resulting in 50% biomass reduction (ED50) 13 days after treatment in the populations WUS, ACR, MCR, and TSR in response to treatment with atrazine (ATZ), amicarbazone (AMI), metribuzin (METRI), or the amicarbazone/metribuzin mix (AMI + METRI). X field rate denotes the herbicide dose expressed as a multiple of the recommended field rate (1X). The rate listed for the mixture (1.75:1 amicarbazone:metribuzin) reflects the rate for amicarbazone only. Relative potencies (RP) are calculated by dividing the ED50 of atrazine by the ED50 of the herbicide in each population, using ED50 values relative to the field rate. Table S3. Structural assessment metrics for the 3‐D molecular models of Amaranthus tuberculatus PSII D1 and GSTF2 proteins. Table S4. Cis‐acting regulatory elements (CARE) identified in the promoter region of the Amaranthus tuberculatus AtuGSTF2 gene. Table S5. Summary of predicted substrate binding pockets at the active sites of the Amaranthus tuberculatus PSII D1 and GSTF2 protein homology models. Residues overlapping between predicted pockets for the AtuGSTF2 model are indicated in bold. Fig. S1. Atrazine resistance prescreening across WUS, ACR, MCR, and TSR populations. (A) In‐silico PCR and amplicon restriction enzyme digestion showing the expected target‐site resistance assay band [file PS-82-6864-s001.docx]

**Supplementary Information**

**TABLES**

**Table S1.** Effective dose estimates resulting in 20% biomass reduction (ED_20_) 13 days after treatment (DAT) in the populations WUS, ACR, MCR, and TSR in response to treatment with atrazine (ATZ), amicarbazone (AMI), metribuzin (METRI), or the amicarbazone/metribuzin mix (AMI+METRI). X field rate denotes the herbicide dose expressed as a multiple of the recommended field rate (1X). The rate listed for the mixture (1.75:1 amicarbazone:metribuzin) reflects the rate for amicarbazone. Relative potencies (RP) are calculated by dividing the ED_20_ of atrazine by the ED_20_ of the herbicide in each population, using ED_20_ values relative to the field rate.

| Population | Herbicide | ED_20_ (95% CI) | ED_20_ (95% CI) | RP (95% CI) | P-value |
| --- | --- | --- | --- | --- | --- |
|  |  | ——g ai ha^-1^—— | ——X Field rate—— |  |  |
| WUS | ATZ | 0.767 (-1.03, 2.56) | 4.57e-04 (-6.12e-04, 1.53e-03) | — | — |
|  | AMI | 0.171 (-0.173, 0.516) | 3.42e-04 (-3.46e-04, 1.03e-03) | 1.33 (-2.77, 5.43) | 0.875 |
|  | METRI | 0.334 (-5.81e-2, 0.726) | 1.17e-03 (-2.03e-04, 2.55e-03) | 0.389 (-0.629, 1.41) | 0.238 |
|  | AMI + METRI | 0.257 (-0.339, 0.853) | 5.13e-04 (-6.77e-04, 1.70e-03) | 0.887 (-2.03, 3.81) | 0.939 |
| ACR | ATZ | 2.26 (-1.76, 6.29) | 1.35e-03 (-1.05e-03, 3.74e-03) | — | — |
|  | AMI | 0.484 (-0.026, 0.994) | 9.67e-04 (-5.09e-05, 1.99e-03) | 1.39 (-1.48, 4.27) | 0.789 |
|  | METRI | 1.36 (-0.633, 3.36) | 4.77e-03 (-2.22e-03, 1.18e-02) | 0.282 (-0.368, 0.932) | **0.030** |
|  | AMI + METRI | 0.158 (-0.164, 0.480) | 3.15e-04 (-3.28e-04, 9.59e-04) | 4.26 (-7.27, 15.8) | 0.578 |
| MCR | ATZ | 8.42 (-15.3, 32.2) | 5.01e-03 (-9.13e-03, 1.92e-02) | — | — |
|  | AMI | 0.257 (-0.531, 1.04) | 5.13e-04 (-1.06e-03, 2.08e-03) | 9.93 (-31.4, 51.3) | 0.671 |
|  | METRI | 0.996 (-2.10, 4.10) | 3.50e-03 (-7.38e-03, 1.44e-02) | 1.44 (-4.62, 7.50) | 0.886 |
|  | AMI + METRI | 0.143 (-0.264, 0.549) | 2.85e-04 (-5.26e-04, 1.10e-03) | 17.9 (-53.9, 89.7) | 0.644 |
| TSR | ATZ | 5.93 (-33.3, 45.2) | 3.53e-03 (-1.98e-02, 2.69e-02) | — | — |
|  | AMI | 104 (-377, 584) | 0.207 (-0.753, 1.17) | 2.54e-02 (-0.178, 0.228) | **<0.001** |
|  | METRI | 179 (-294, 652) | 0.629 (-1.03, 2.29) | 6.26e-03 (-3.76e-02, 5.02e-02) | **<0.001** |
|  | AMI + METRI | 0.818 (-3.92, 5.56) | 1.63e-03 (-7.82e-03, 1.11e-02) | 2.67 (-21.2, 26.5) | 0.890 |

^†^Relative potencies significantly different from 1 are indicated in bold (α=0.05)

**Table S2.** Effective dose estimates resulting in 50% biomass reduction (ED_50_) 13 days after treatment in the populations WUS, ACR, MCR, and TSR in response to treatment with atrazine (ATZ), amicarbazone (AMI), metribuzin (METRI), or the amicarbazone/metribuzin mix (AMI+METRI). X field rate denotes the herbicide dose expressed as a multiple of the recommended field rate (1X). The rate listed for the mixture (1.75:1 amicarbazone:metribuzin) reflects the rate for amicarbazone only. Relative potencies (RP) are calculated by dividing the ED_50_ of atrazine by the ED_50_ of the herbicide in each population, using ED_50_ values relative to the field rate.

| Population | Herbicide | ED_50_ (95% CI) | ED_50_ (95% CI) | RP (95% CI) | P-value |
| --- | --- | --- | --- | --- | --- |
|  |  | ——g ai ha^-1^—— | ——X Field rate—— |  |  |
| WUS | ATZ | 10.6 (-2.11, 23.3) | 6.29e-03 (-1.26e-03, 1.38e-02) | — | — |
|  | AMI | 1.84 (-0.256, 3.93) | 3.66e-03 (-5.11e-04, 7.84e-03) | 1.72 (-1.12, 4.56) | 0.620 |
|  | METRI | 1.75 (0.519, 2.98) | 6.14e-03 (1.82e-03, 1.05e-02) | 1.03 (-0.401, 2.45) | 0.972 |
|  | AMI+METRI | 1.07 (-0.112, 2.25) | 2.13e-03 (-2.23e-04, 4.49e-03) | 2.95 (-1.85, 7.75) | 0.426 |
| ACR | ATZ | 29.2 (-0.103, 58.6) | 1.74e-02 (-6.15e-05, 3.48e-02) | — | — |
|  | AMI | 2.80 (1.07, 4.53) | 5.58e-03 (2.13e-03, 9.04e-03) | 3.11 (-0.558, 6.78) | 0.258 |
|  | METRI | 10.6 (1.92, 19.2) | 3.70e-02 (6.73e-03, 6.73e-02) | 0.469 (-0.138, 1.08) | 0.087 |
|  | AMI+METRI | 1.42 (-1.11e-2, 2.85) | 2.84e-03 (-2.13e-05, 5.70e-03) | 6.12 (-2.58, 14.8) | 0.248 |
| MCR | ATZ | 228 (-137, 593) | 0.136 (-8.18e-02, 0.353) | — | — |
|  | AMI | 5.71 (-4.31, 15.7) | 1.14e-02 (-8.60e-03, 3.14e-02) | 12.0 (-16.6, 40.6) | 0.448 |
|  | METRI | 19.5 (-13.4, 52.5) | 6.85e-02 (-4.71e-02, 0.184) | 1.98 (-2.64, 6.60) | 0.677 |
|  | AMI+METRI | 1.90 (-0.969, 4.77) | 3.80e-03 (-1.94e-03, 9.53e-03) | 36.1 (-43.5, 116) | 0.386 |
| TSR | ATZ | 2.30e+3 (-2.90e+3, 7.50e+3) | 1.37 (-1.72, 4.46) | — | — |
|  | AMI | 2.42e+3 (-2.59e+3, 7.42e+3) | 4.83 (-5.17, 14.8) | 0.278 (-0.745, 1.30) | 0.166 |
|  | METRI | 740 (-90.3, 1.57e+3) | 2.60 (-0.317, 5.51) | 0.553 (-0.863, 1.97) | 0.534 |
|  | AMI+METRI | 83.2 (-142, 308) | 0.166 (-0.283, 0.615) | 8.89 (-23.8, 41.6) | 0.635 |

**Table S3.** Structural assessment metrics for the 3-D molecular models of *Amaranthus tuberculatus* PSII D1 and GSTF2 proteins.

| Model | Template | Cα RMSD^†^ | Sequence identity (%) | GMQE^‡^ | QMEANDisCo global^§^ | Ramachandran favored (%)^¶^ |
| --- | --- | --- | --- | --- | --- | --- |
| PSII D1 | 4V82 | 0.106 | 87.76 | 0.82 | 0.75 | 95.20 |
| AtuGSTF2 | 1BX9 | 0.137 | 54.59 | 0.82 | 0.80 | 97.07 |

^†^Cα RMSD, root mean square deviation between alpha carbon atom positions of template and model

^‡^GMQE, Global Model Quality Estimate of the template

^§^QMEANDisCo global, Distance Constraint Qualitative Model Energy Analysis global score of the model

^¶^Percentage of model residues in Ramachandran favored regions

**Table S4.** Cis-acting regulatory elements (CARE) identified in the promoter region of the *Amaranthus tuberculatus* *AtuGSTF2* gene.

| CARE | Motif | Position | Score | Strand | Species | Description |
| --- | --- | --- | --- | --- | --- | --- |
| ABRE | ACGTG | 509 | 5 | + | Arabidopsis thaliana | cis-acting element involved in the abscisic acid responsiveness |
| WRE3 | CCACCT | 692 | 6 | + | Pisum sativum | NA |
| as-1 | TGACG | 108 | 5 | + | Arabidopsis thaliana | NA |
| CAAT-box | CAAAT | 83 | 5 | - | Pisum sativum | common cis-acting element in promoter and enhancer regions |
| CAAT-box | CAAAT | 105 | 5 | - | Pisum sativum | common cis-acting element in promoter and enhancer regions |
| CAAT-box | CAAAT | 270 | 5 | + | Pisum sativum | common cis-acting element in promoter and enhancer regions |
| CAAT-box | CAAT | 308 | 4 | + | Nicotiana glutinosa | NA |
| CAAT-box | CAAT | 374 | 4 | + | Nicotiana glutinosa | NA |
| CAAT-box | CCAAT | 376 | 5 | - | Arabidopsis thaliana | common cis-acting element in promoter and enhancer regions |
| CAAT-box | CAAAT | 436 | 5 | - | Pisum sativum | common cis-acting element in promoter and enhancer regions |
| CAAT-box | CAAAT | 535 | 5 | - | Pisum sativum | common cis-acting element in promoter and enhancer regions |
| CAAT-box | CAAAT | 567 | 5 | - | Pisum sativum | common cis-acting element in promoter and enhancer regions |
| CAAT-box | CAAT | 648 | 4 | - | Nicotiana glutinosa | NA |
| CAAT-box | CAAAT | 656 | 5 | - | Pisum sativum | common cis-acting element in promoter and enhancer regions |
| CAAT-box | CAAAT | 669 | 5 | + | Pisum sativum | common cis-acting element in promoter and enhancer regions |
| CAAT-box | CCAAT | 682 | 5 | - | Arabidopsis thaliana | common cis-acting element in promoter and enhancer regions |
| CAAT-box | CAAAT | 718 | 5 | + | Pisum sativum | common cis-acting element in promoter and enhancer regions |
| CAAT-box | CAAT | 741 | 4 | + | Nicotiana glutinosa | NA |
| CAAT-box | CAAT | 792 | 4 | + | Nicotiana glutinosa | NA |
| CAAT-box | CAAT | 829 | 4 | - | Nicotiana glutinosa | NA |
| CAAT-box | CAAT | 957 | 4 | - | Nicotiana glutinosa | NA |
| CAAT-box | CAAAT | 986 | 5 | - | Pisum sativum | common cis-acting element in promoter and enhancer regions |
| G-Box | CACGTT | 508 | 6 | - | Pisum sativum | cis-acting regulatory element involved in light responsiveness |
| Box 4 | ATTAAT | 912 | 6 | - | Petroselinum crispum | part of a conserved DNA module involved in light responsiveness |
| CGTCA-motif | CGTCA | 108 | 5 | - | Hordeum vulgare | cis-acting regulatory element involved in the MeJA-responsiveness |
| I-box | GATAAGGGT | 153 | 9 | + | Arabidopsis thaliana | part of a light responsive element |
| I-box | atGATAAGGTC | 518 | 10 | - | Helianthus annuus | part of a light responsive element |
| TATA | TATAAAAT | 934 | 8 | - | Arabidopsis thaliana | NA |
| TGACG-motif | TGACG | 108 | 5 | + | Hordeum vulgare | cis-acting regulatory element involved in the MeJA-responsiveness |
| AT~TATA-box | TATATAAA | 936 | 8 | - | Arabidopsis thaliana | NA |
| AT~TATA-box | TATATA | 938 | 6 | - | Arabidopsis thaliana | NA |
| STRE | AGGGG | 354 | 5 | - | Arabidopsis thaliana | NA |
| GT1-motif | GGTTAA | 264 | 6 | - | Arabidopsis thaliana | light responsive element |
| MYC | CATGTG | 62 | 6 | + | Arabidopsis thaliana | NA |
| MYC | CATGTG | 326 | 6 | + | Arabidopsis thaliana | NA |
| MYC | CAATTG | 374 | 6 | + | Arabidopsis thaliana | NA |
| MYC | CATTTG | 655 | 6 | + | Arabidopsis thaliana | NA |
| MYC | CATGTG | 710 | 6 | - | Arabidopsis thaliana | NA |
| MYC | CATGTG | 712 | 6 | + | Arabidopsis thaliana | NA |
| MYC | CATTTG | 985 | 6 | + | Arabidopsis thaliana | NA |
| TATA-box | ATATAA | 273 | 6 | + | Brassica oleracea | core promoter element around -30 of transcription start |
| TATA-box | TATA | 274 | 4 | + | Arabidopsis thaliana | core promoter element around -30 of transcription start |
| TATA-box | TATACA | 814 | 6 | - | Helianthus annuus | core promoter element around -30 of transcription start |
| TATA-box | TATA | 816 | 4 | - | Arabidopsis thaliana | core promoter element around -30 of transcription start |
| TATA-box | ATATAT | 891 | 6 | - | Brassica napus | core promoter element around -30 of transcription start |
| TATA-box | TATA | 892 | 4 | - | Arabidopsis thaliana | core promoter element around -30 of transcription start |
| TATA-box | ATTATA | 901 | 6 | + | Brassica napus | core promoter element around -30 of transcription start |
| TATA-box | TATAA | 902 | 5 | - | Arabidopsis thaliana | core promoter element around -30 of transcription start |
| TATA-box | TATA | 903 | 4 | - | Arabidopsis thaliana | core promoter element around -30 of transcription start |
| TATA-box | TATTTAAA | 905 | 8 | + | Arabidopsis thaliana | core promoter element around -30 of transcription start |
| TATA-box | TATAAAA | 935 | 7 | - | Pisum sativum | core promoter element around -30 of transcription start |
| TATA-box | TATAAA | 936 | 6 | - | Helianthus annuus | core promoter element around -30 of transcription start |
| TATA-box | TATATAA | 937 | 7 | - | Arabidopsis thaliana | core promoter element around -30 of transcription start |
| TATA-box | TATATA | 938 | 6 | - | Arabidopsis thaliana | core promoter element around -30 of transcription start |
| TATA-box | ATATAA | 939 | 6 | + | Brassica oleracea | core promoter element around -30 of transcription start |
| TATA-box | TATA | 940 | 4 | - | Arabidopsis thaliana | core promoter element around -30 of transcription start |
| MRE | AACCTAA | 775 | 7 | + | Petroselinum crispum | MYB binding site involved in light responsiveness |

**Table S5.** Summary of predicted substrate binding pockets at the active sites of the *Amaranthus tuberculatus* PSII D1 and GSTF2 protein homology models. Residues overlapping between predicted pockets for the AtuGSTF2 model are indicated in bold.

| Model | Number of residues | Pocket volume (Å^3^) | Probability | Residues |
| --- | --- | --- | --- | --- |
| PSII D1 | 17 | 976.3 | 0.592 | F211, M214, H215, L218, V219, Y246, I248, A251, H252, F255, A263, S264, F265, S268, L271, F274, L275 |
| AtuGSTF2 | 16 | 735.6 | 0.306 | I10, S11, G12, **C13**, R16, **Q53**, **V54**, **E108**, A111, **I112**, A115, M116, Y120, P171, A172, T175 |
|  | 10 | 406.0 | 0.154 | **C13**, N49, **Q53**, **V54**, P55, E66, S67, R68, **E108**, **I112** |

**FIGURES**


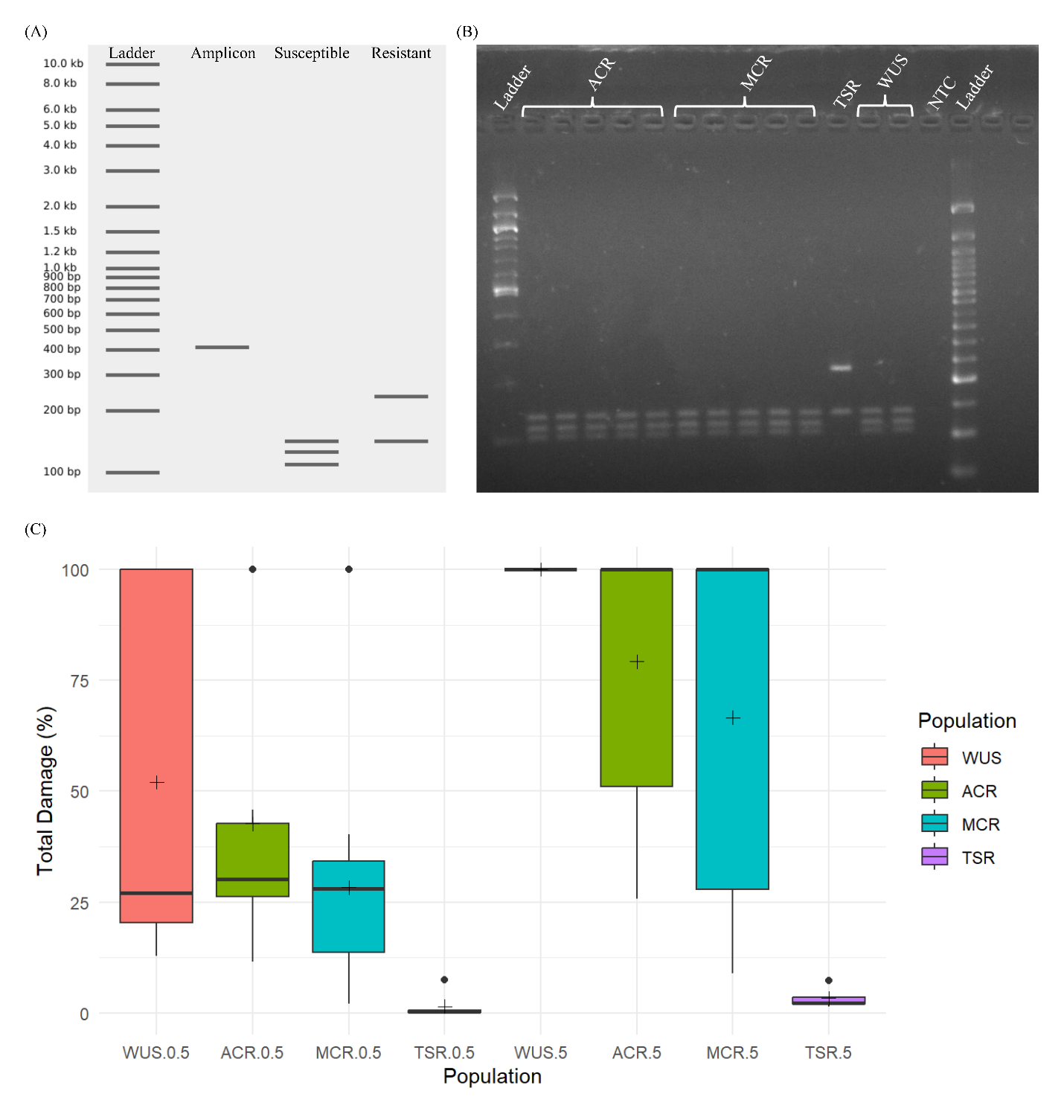


**Figure S1.** Atrazine resistance prescreening across WUS, ACR, MCR, and TSR populations. (A) In-silico PCR and amplicon restriction enzyme digestion showing the expected target-site resistance assay banding patterns for the Ser^264^Gly mutation. (B) Agarose gel-electrophoresis showing the target-site resistance assay banding patterns observed in the populations evaluated; on the left is a 100-bp ladder and on the right is a 50-bp ladder. (C) Total damage relative to the control in response to atrazine applied POST at 0.5X (left) and 5X field rates (right).


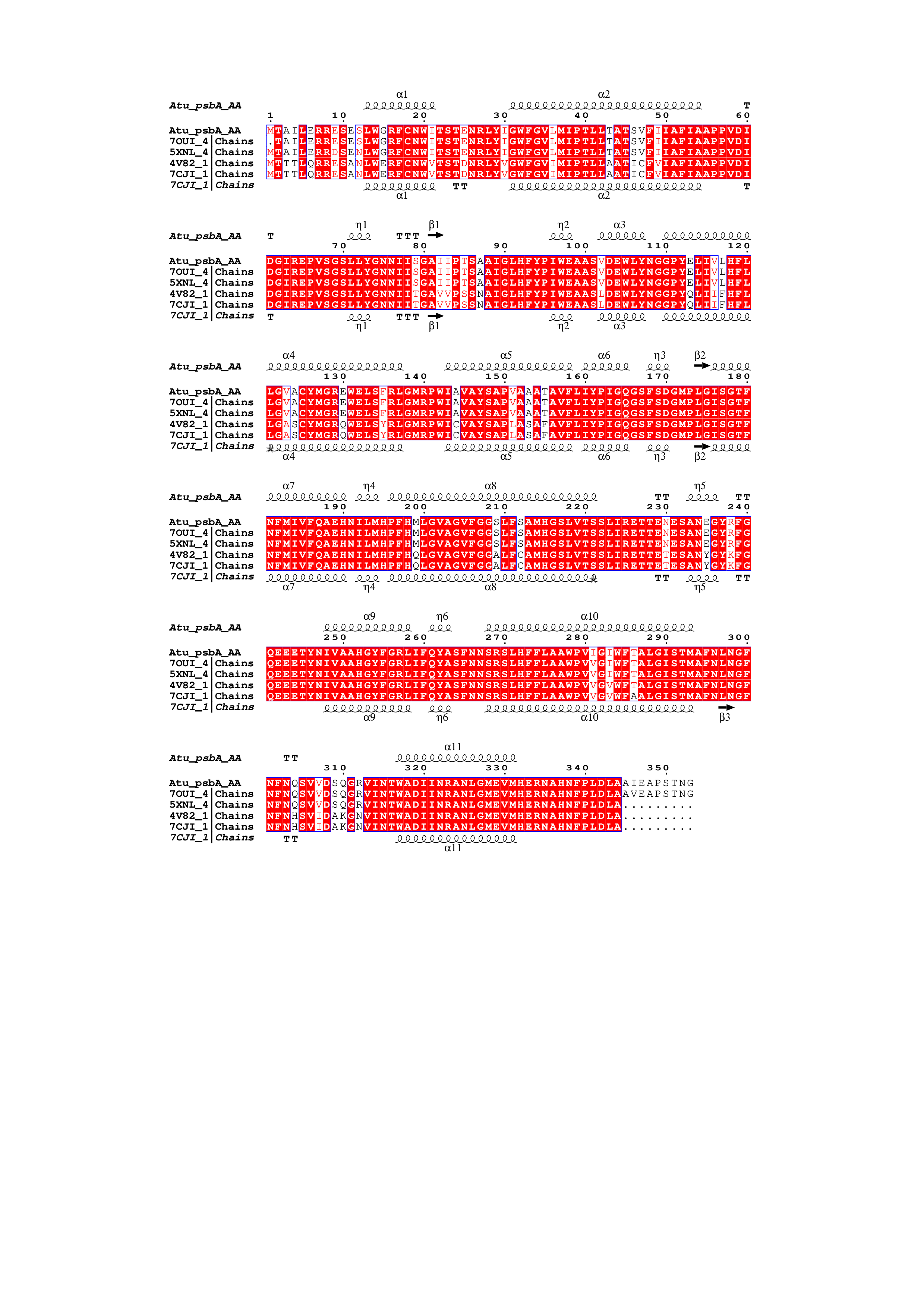


**Figure S2.** *Amaranthus tuberculatus* PSII D1 protein multiple sequence alignment against homologous proteins from *Arabidopsis thaliana* (7OUI), *Pisum sativum* (5XNL), *Thermosynechococcus elongatus* (4V82), and *Thermostichus vulcanus* (7CJI). Stretches of conserved residues are enclosed in a blue box, with strictly conserved (identical) residues shown in white with a red background and functionally conserved (similar properties) residues shown in red with no background; non-conserved residues are shown in black with no background. Secondary structure of the predicted model and template model are shown above and below the alignment, respectively, and labeled with the type of structure: α (alpha helix), η (short alpha helix), β (beta sheet), and T (turn).


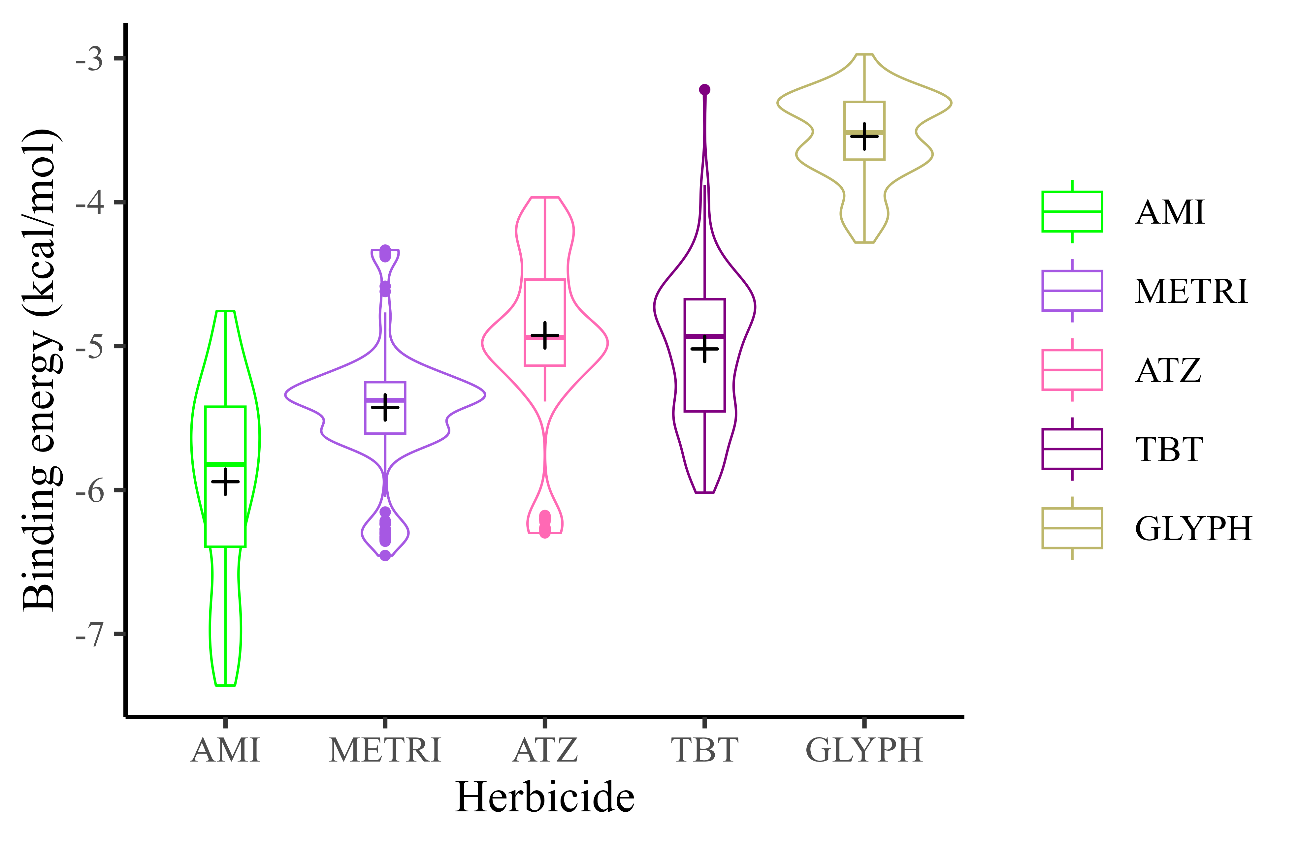


**Figure S3.** Distribution of predicted binding energies (kcal/mol) reported across all 20 docking simulations for each herbicide docked within the Q_B_ pocket of the *Amaranthus tuberculatus* PSII D1 protein homology model: amicarbazone (AMI), metribuzin (METRI), atrazine (ATZ), terbutryn (TBT), glyphosate (GLYPH).


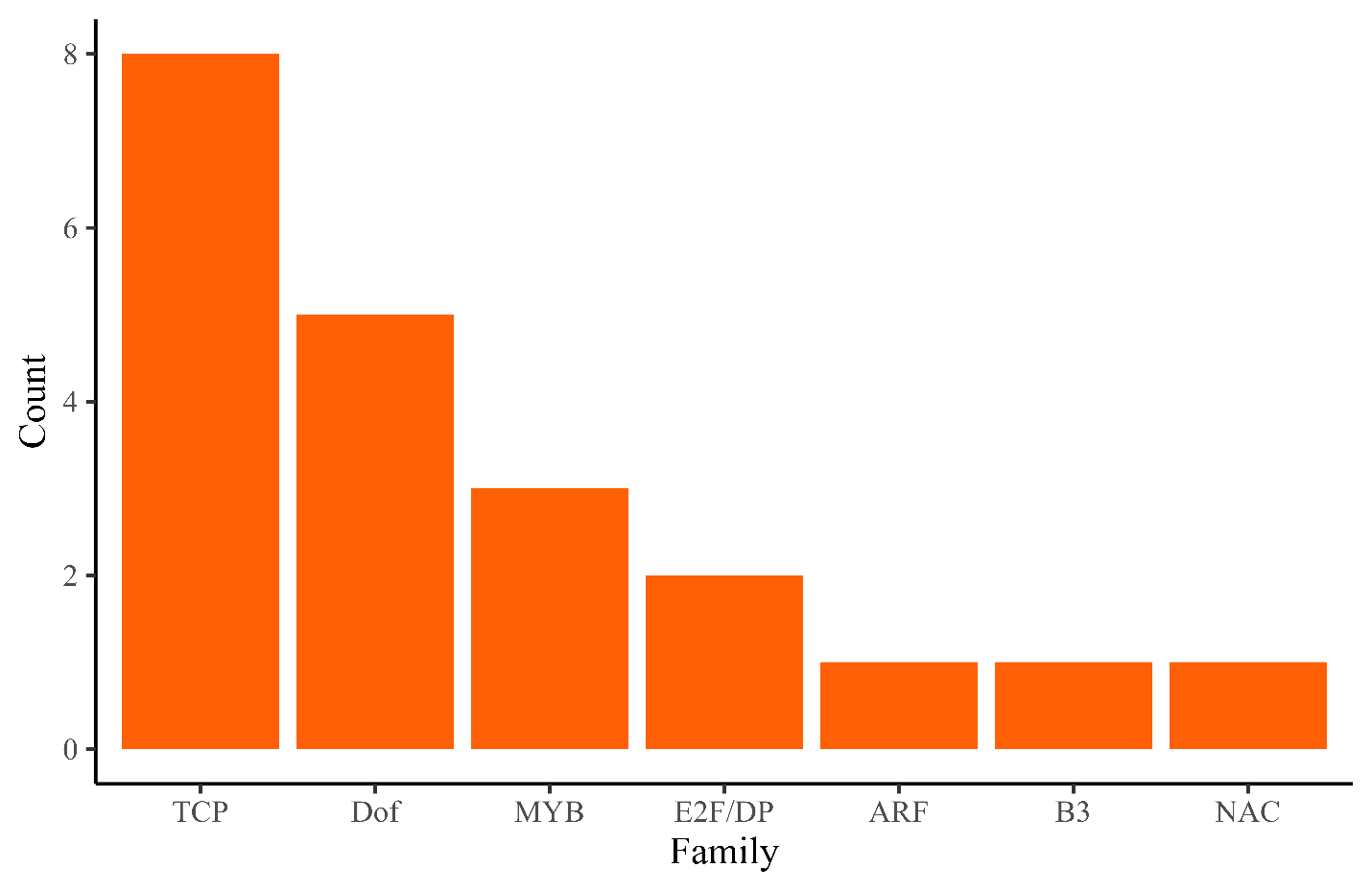


**Figure S4.** The number of transcription factors (TF) belonging to different TF families predicted to have potential regulatory relationships with *AtuGSTF2*.


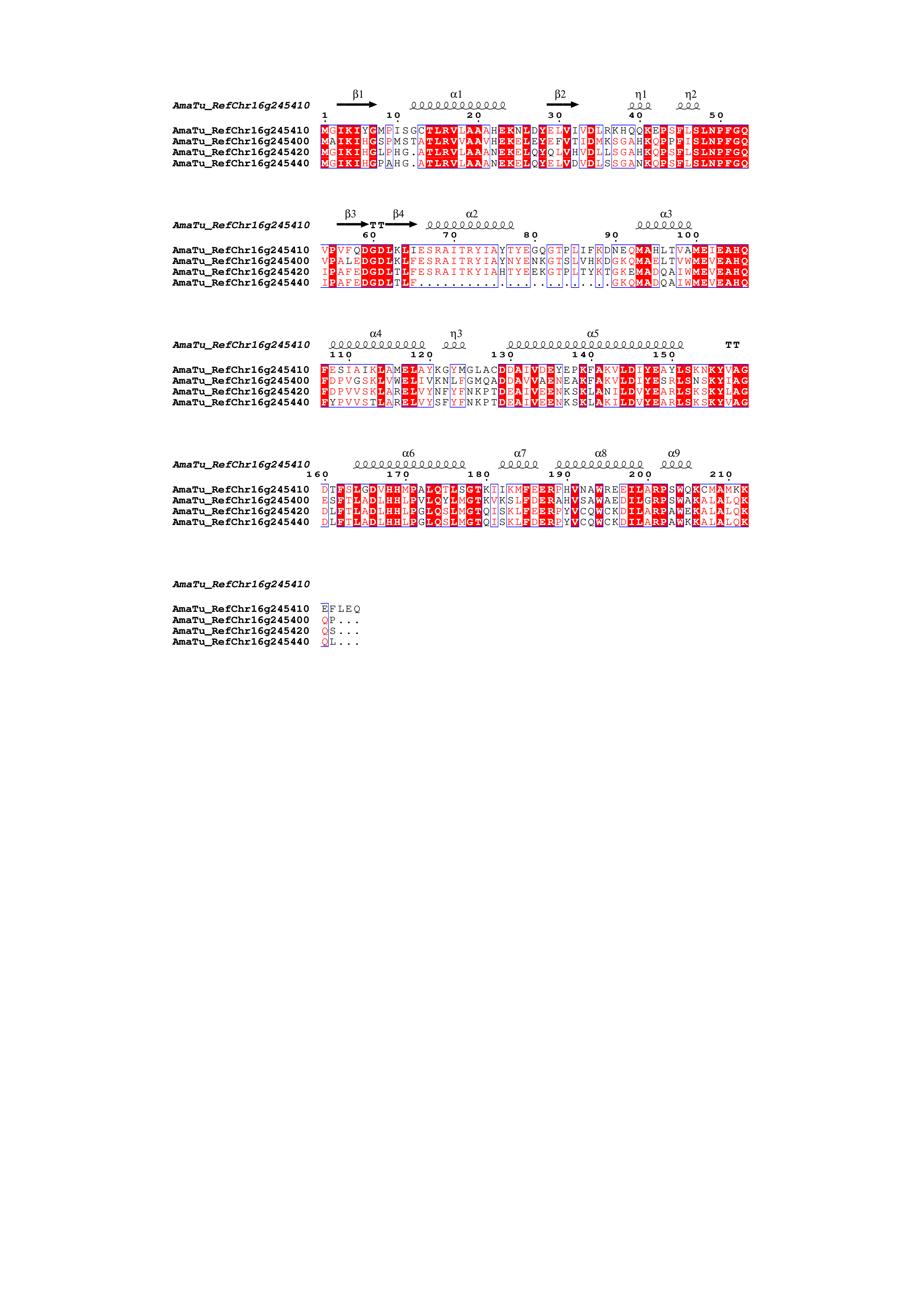


**Figure S5.** *Amaranthus tuberculatus* AtuGSTF2 (AmaTuRefChr16g245410) protein multiple sequence alignment against the three GSTs (AmaTuRefChr16g245400, AmaTuRefChr16g245420, and AmaTuRefChr16g245440) identified within 30-kb of AtuGSTF2 on chromosome 16. Stretches of conserved residues are enclosed in a blue box, with strictly conserved (identical) residues shown in white with a red background and functionally conserved (similar properties) residues shown in red with no background; non-conserved residues are shown in black with no background. Secondary structure of the predicted model and template model are shown above and below the alignment, respectively, and labeled with the type of structure: α (alpha helix), η (short alpha helix), β (beta sheet), and T (turn).
